# Biomarkers reflecting the pathogenesis, clinical manifestations, and guide therapeutic approach in systemic sclerosis: a narrative review

**DOI:** 10.1007/s10067-024-07123-y

**Published:** 2024-08-29

**Authors:** Anna Bazsó, Péter Szodoray, Yehuda Shoenfeld, Emese Kiss

**Affiliations:** 1 Department of Clinical Immunology, Adult and Paediatric Rheumatology, National Institute of Locomotor System Disorders and Disabilities, Budapest, Hungary; 2 Department of Immunology, Oslo University Hospital, Rikshospitalet and University of Oslo, Oslo, Norway; 3 Reichmann University, Herzelia, Israel; 4 Zabludowicz Center for Autoimmune Diseases, Sheba Medical Center, 5265601 Tel-Hashomer, Israel; 5 Division of Locomotor System and Rheumatology Prevention, Department of Internal Medicine and Haematology, Semmelweis University, Budapest, Hungary

**Keywords:** Biomarkers, Organ manifestations, Prognosis, Systemic sclerosis

## Abstract

Systemic sclerosis (SSc) is a progressive autoimmune disorder that mainly affects the skin. There are other clinical manifestations as renal, pulmonary, cardiovascular, and gastrointestinal tract involvements. Based on the skin involvement there are two subtypes of SSc, as limited cutaneous SSc (lSSc) which involves the acral part of the body and diffuse cutaneous SSc (dSSc) resulting in significant skin thickening of the body. Despite of the extensive research the pathomechanism is not fully clarified, how Ssc develops, moreover identifying biomarkers to predict the clinical outcome and prognosis still remains challenging. Circulating biomarkers can be crucial to define the diagnosis, to predict the prognosis and monitor the clinical course. However, only some patients are responsive to the therapy in SSc, and there is a need to reach the ideal therapy for any individual to prevent or slow down the progression in early stages of the disease. In this narrative review, our purpose was to summarize the potential biomarkers in Ssc, describe their role in the diagnosis, pathomechanism, clinical course, organ manifestations, as well as the response to the therapy. Biomarkers assessment aids in the evaluation of disease progression, and disease outcome.

## Introduction

Systemic sclerosis (Ssc) is a multiorgan autoimmune disease with cutaneous and organ manifestations. Ssc is characterized by vascular abnormalities, humoral and cellular immune disturbances, and extensive skin and organ fibrotic processes. Despite of the expanding knowledge of the defective immune mechanism in the background, to predict the clinical course and outcome of the treatment of Ssc faces difficulties. The dysregulated immune system could facilitate the development of organ manifestations through impaired vascularity and lead to chronic inflammation and irreversible fibrosis. Predictive molecular and cellular tools—as biomarkers—are needed to measure, investigate, and evaluate the development of the disease, pathologic pathways, and pharmacologic responses to the treatment. Therefore, biomarkers as “indicators” have a very strong predictive, diagnostic, and prognostic role. There are several concepts to classify the biomarkers in the systemic diseases. Reviews classify the biomarkers based on their molecular functions, while others focus on their role in the pathological processes.

Our aim was to analyze the variability of the biomarkers in Ssc, identify their entity, and facilitate to understand their state, role, and importance in the diagnostics and disease outcome evaluation. Based on the diversity of the biomarkers, it is not easy to establish a strong and clear biomarker hyrarchy in the diagnosis, follow-up, or outcome of the disease for clinicians.

We aimed to establish a hierarchy of the biomarkers in Ssc based on their function in the (1) immune system, (2) pathogenesis, and (3) clinical course, organ manifestation, and disease activity.

## The pathomechanism and progressivity of systemic sclerosis

Several environmental and genetic interactions predispose to the appearance of the disease. Besides the cellular and humoral immune abnormalities, inflammatory cytokines, the distortion in the balance of the growth factors and the autoantibodies result to the fibroproliferative vasculopathy and finally, the cutaneous and visceral fibrosis [1]. However, there is a complex cascade which results in vascular injury and fibrotic process in Ssc, three pathways caused by immunological alteration and phenotypical manifestations, such as (1) vascular abnormalities, (2) autoimmune/immune attacks, and (3) fibrosis.

The importance of the genetic and epigenetic background is continuously increasing. The HLA genes in the SSc pathomechanism are proved to have the strongest association with the antibodies and predisposing factors [2]. There are some differences between the African-American (HLA-DRB1*08.04, HLA-DRB1*11.02) and European-American (DPB1*13.01, HLA-DRB1*07.01) cohorts. While the HLA-DRB1*08.04, HLA-DRB1*11.02 alleles are associated with the development of SSc, the HLA-DRB1*11.02 alleles are related to the anti-fibrillarin antibody onset. In the European-American cohort, the DPB1*13.01, HLA-DRB1*07.01 refers to anti-topoisomerase-1 (ATA) and anti-centromere (ACA) antibodies [3]. In the Ssc myofibroblast and non-myofiboblasts, the neuroblastoma breakpoint family (NBPF) genes are highly expressed. The mutations of potassium channel genes—KCNK5, ABCC—are related to the PAH in Ssc [4]. The pathogenic association in Ssc could be grouped in three major pathways, such as genes, associated with vascularization (eNOS, ACA, ET-1, ETR-A/B), immune or inflammatory genes (STAT4, IRF5, CD247), and genes associated with fibrotic processes (MIF, CTGF, Fibrillin-1, SPARC) [5].

Moreover, there are many other tissue-specific transcription factors (ELF1, MGA) are overexpressed while KLF4 and ID4 are downregulated in Ssc blood cells [6]. Zou et al. have studied chromosome regions of SSc, and their findings proved the number genetic loci were associated with high prevalence of Choctaw Indians [7].

The molecular mimicri hypothesis is also supported as homologous sequences of the autoantibodies of SSc and the viral proteins (Mimiviridae and Pycodnaviridae families) [3].

The other important molecular pathway is the epigenetic modifications, which lead to the pathognomic molecular alteration in the fibroblasts and drive the activation of profibrotic factors (HOTAIR/EZH2/NOTCH) by mi-RNA-34a. The abnormality of the chromatin tools of dendritic cells has a prominent and accountable role in the epigenetic process in Ssc patients [8].

Both the innate and adaptive immune system have a significant impact on the pathogenesis of Ssc. Among others, type 1 interferon (T1 IFN), fibroblast growth factors (FGFs), and its receptors (FGFRs) contribute to the profibrotic process by the FGF9/FGFR3 abnormality [9].

Interaction of the genetic predisposition and environmental stimuli (viruses, organic solvents, oxidative stress, autoantibodies) triggers the immune cells’ activation, phenotypical alteration of vascular cells, and fibroblasts.

Distinct, crucial steps in the pathomechanism are hallmarks of the progressivity of Ssc. In the local microvascular functional dysregulation, the microvascular damage can be persistent. Various autoimmune processes, fibrotic mechanisms, and related tissue hypoxia lead to systemic fibrosis [10].

Endothelial cells are activated and undergo structural changes. Behind the vascular damage, angiogenesis is also a crucial step in the vasculopathy in SSc, and vasculogenesis is a defective alteration driven by pro-angiogenetic factors and lack of anti-angiogenic factors. These vascular structural abnormalities are catalyzed by adhesion molecules and associated with tissue hypoxia. Also, the imbalance between vasoconstriction and vasodilatation is due to vascular damage and hypercoagulation by enhanced expression of specific molecules (endothelin) and suppressed amount and function of prostacyclin and nitric oxides, among others [11].

Altogether, there are six morphological features of the microvascular patterns, driven by tissue-specific molecules and autoantibodies. In the very early pattern, only some microvascular alteration can be detected. Later, in the attraction of fibrotic elements and transmigration of inflammatory cells, growth factors lead to the increased microvascular damage and tissue fibrosis (early and early-active phase). In the remarkable active phase, a complex fibroproliferative and occlusive interaction of inflammatory and autoimmune elements is identified. In the late phase, driven by tissue hypoxia and microvascular damage, the extensive fibrosis is the prominent feature of SSc [10, 12].

In the early phase of Ssc, cell adhesion is often stimulated by activated progenitor cells and increased expression of adhesion molecules. The increased release of growth factors results in cell migrations and platelet aggregations which are related to the structural changes of the vascularity and results in increased permeability and giant capillarity with hemorrhages and edema [13].

In the immune or active phase, more extensive activation of the innate and adaptive immune system, proinflammatory cytokines, increased cell death, adhesion molecules, and damage-associated molecular patterns altogether lead to the vascular damage.

The activation of endothelial cells through the endothelin-1 and chemokines stimulates the inflammatory cells, the inflammation cascade. The impaired balance of Th17/Treg cells and Th2 cell dominancy triggers a chain of inflammatory sequelae and the overproduction of inflammatory cytokines (IL-8, IL-4, IL-13, CCL2, MMP-1) [14].

On the contrary, the anti-inflammatory responses are mainly reduced in Ssc. Lower percentages of regulatory T cells, regulatory B cells, natural killer cells (NK-cells), and reduced interleukin-10 (IL-10) secretion are observed in Ssc [1, 10–15].

The molecular and cellular dysregulation leads to endothelial cell activation, vascular occlusion, vasculogenesis, and tissue hypoxia by fibroblasts, T and B cells, and endothelial cell activity. IL-4 and IL-13 induce B cell proliferation leading to the production of immunglobulins, adhesion molecules, and inflammatory cytokines [16].

Besides T cell abnormalities, B cells also contribute to the progression of Ssc. B cells secrete IL-6, which became one of the most relevant therapeutic targets. The presence of specific autoantibodies—which can be present in most of the SSc patients—is also a strong evidence that B cells play an important pathogenic role. The dysregulation and abnormal function of B cells also represent in the clinical manifestation. The activation and antibody production of B cells promote further cytokine and macrophage activation and correlate the disease progression and contribute in both the vascular and fibrotic phase of the disease [15, 16].

Dendritic cells (DCs) also have a critical pathognomonic role in the Ssc pathophysiology. DCs contribute to antigen presentation and activate naïve T cells. Interferon-ɑ (IFN-ɑ), chemokine ligand 4 (CXCL4) secretion is stimulated by toll-like receptor-8 (TLR8). TLR8 is expressed by plasmacytoid DCs (pDCs) and enhances the profibrotic processes in the skin. IFN-ɑ is promoted by pDCs and correlates the development and progression of Ssc [17].

The overstimulation of monocytes, M2 macrophages, mast cells, and therefore the excessive TGFß, IL-4, IL-6, IL-13, platelet-derived growth factor (PDGF), TNF-ɑ production stimulates directly other profibrotic factors and chemoattractive and intercellular adhesion molecules. However, a wide spectrum of pro-inflammatory cells can be detected in these inflammatory processes. DCs, monocytes, M2 macrophages, mast cells, and type 2 helper cells (Th2) contribute mostly in the early phase of the inflammation. IL-4 and IL-13 produced by Th2 cells activate macrophages and fibroblasts to produce TGFß, as well [18, 19].

The obliterative vasculopathy and the fibroblast activation are connected strongly by the immune cells and cytokines mentioned above.

In the late phase of Ssc, the fibrotic processes, increased TGFß production results in collagen synthesis and fibroblast proliferation. Activation of the circulating fibrocytes could migrate from the bloodstream and accumulate in the surrounding tissue. On the other side, the inflammatory cascade directly inhibits the anti-inflammatory factors, as the synthesis of metalloproteinase 1 and 3 (MMP1, MMP3) [1, 18, 20]. Despite of the prominent role in late phase, the TGFß could be elevated in the early, active phase of the disease, especially in the skin, as well. TGFß activates the proinflammatory cytokines and regulates adhesion molecules, however; in the late phase, it activates or dysregulates the normal fibroblasts. In fibroblast activation, resident fibroblasts, pre-adipocytes, endothelial cells, mesenchymal stem cells, and fibrocytes trans-differentiate by activation through TGFß. As a result of the transactivation, myofibroblasts are activated, and further pro-inflammatory cytokines are secreted rapidly and continuously [21]. Myofibroblasts are the source of the main extracellular matrix elements such as elastin, collagens, fibronectin, and proteoglycans. The presence of myofibroblasts is not specific but prognostic for connective tissue diseases, especially for SSc. The loss of normal apoptosis of the immune cells is also a key process in the development of SSc. Therefore, the abnormally activated myofibroblasts survive which results in prolonged fibrosis and increased rigidity of the tissues [22].

Taking together, from the tissue injury and vasculopathy to the fibrosis, the inflammation and autoimmune processes could not be easily distinctive as fibroblasts, and the immune cells maintain the immune response and fibrosis, also. The loss of balance of the vasoconstriction and vasodilatation and the loss of molecular control of angiogenic and angiostatic factors determine the clinical feature and prognosis of Ssc.

## Clinical manifestations and screening tools

The skin involvement is still the hallmark of SSc. The cutaneous involvement defines two forms, such as limited or diffuse cutaneous scleroderma which can be associated with different extent of body rigidity. As the modified Rodnan skin scores (mRSS) gives highly variable results by the clinicians, the high-frequency ultrasound seems to be a more specific and useful tool to detect skin alterations [23, 24].

Musculoskeletal manifestations are strongly connected with the skin involvement. The progression of the disease is associated with the hand, foot, and further the elbow deformity, and one of the most progressive symptoms, acrosclerosis. SSc and rheumatoid arthritis can overlap in 25% of patients, based on two French studies, and the authors confirmed that the presence and co-existence of rheumatoid factor (RF), anti-citrullinated proteins (ACPA), and anti-carbamylated protein (anti-CarP) antibodies predict a worse prognosis manifested in vascular progression, synovitis, tenosynovitis, digital ulcers (DU), and interstitial lung diseases (ILD) [25, 26].

The neurological manifestations are not rare in this disease. As a result of the derailed immune mechanisms, fibrosis can spread, and both sensory and motor polyneuropathies are observed. Polyneuropathy, trigeminus neuralgia, and mononeuritis multiplex were also reported in a wide range of SSc patients [27].

Vascular abnormalities are very significant symptoms in SSc from the early phase of the disease. These abnormalities are very specific, as well. Raynaud’s phenomenon could be the leading symptom in the early onset and during the progression of the disease too [28]. The worsening of the vasculopathy could manifest in digital ulcers, internal organ involvements as PAH, or malabsorption. Calcinosis is also a specific clinical sign in SSc which is usually reported on the extensor part of the extremities [29]. While the anti-PM/Scl70 antibodies overlap refers a good prognosis, male sex, lower diffusing capacity of lung for carbon monoxide (DLCO < 70%), cardiovascular manifestation, and elevated C-reactive protein (CRP level) (> 5 mg/l) are all reported as indicators for worse outcome [30, 31].

PAH and ILD are still the two main causes of the death in SSc. Regarding vascular abnormalities, mostly arterial stiffness results in hemodynamical changes in the main arterial brunches. Otherwise, pulmonary arterial hypertension (PAH) and inflammatory lung disease (ILD) are characterized by both micro-and macrovascular abnormalities. The DETECT algorithm, echocardiography, and cardiac magnetic resonance imaging (MRI) are potential essential detecting tools in SSc to characterize the stage and phenotype of the cardio-pulmonary manifestation, such as arrythmias, non-ischaemic cardiomyopathy, increased diastolic dysfunction, and myocarditis [32].

SSc-ILD shows different manifestations. Chest x-ray, as well as lung ultrasound, lung density detected by high-resolution computer tomography (HRCT) scan, bronchoalveolar lavage (BAL) can follow disease progression. In BAL fluid (BALF)—which is not routinely performed in Ssc—various biomarkers could be identified. Worsening of pulmonary fibrosis, bronchiectasis, decreased lung diffusing capacity, and the presence of neutrophils in the BAL are also negative prognostic factors [33].

Concerning gastrointestinal (GI) manifestations, esophageal reflux disease, dilatation, and dysmotility have a prominent impact in the prognosis. Transabdominal esophageal ultrasound or manometry usually shows a slower peristalsis or esophageal dilatation [34]. The role of altered gut microbiome has a deep impact in the developing of Ssc and other immune-mediated disorders such as psoriatic arthritis, inflammatory bowel disease (IBD)-related spondyloarthritis, and coeliakia [35]. The dysregulation of the balance of the gut microbiome, such as increased number of Fusobacterium, Ruminococcus, Lactobacillus, and reduced Faecalbacterium can result in the damage of the gut permeability. Moreover, the changes of the gut permeability initiate further immune-mediated or autoimmune responses in the joints and skin, as well. Behind the histopathological assessment by intestinal biopsy which is often complicated to apply, biomarkers could be potent tools to guide us even in the early phase of the disease [36, 37].

### Search strategy

There are several studies which highlighted the importance of different biomarkers in the last decades. However, to evaluate the hierarchy of the biomarkers in SSc is still very challenging both for researchers and physicians. Our concept was to represent and specify the candidate markers of SSc (1) in the immune system, (2) in the disease pathways, and (3) in the organ manifestations or disease activity (Table 1).

**Table 1:** Classification of biomarkers in systemic sclerosis

Classification	Biomarkers
I. The role of biomarkers in the diagnosis system of SSc	
I/1. Autoantibodies (diagnostic)	Anti-Scl-70, anti-CENP-A, anti-Pm-Scl, antifibrillarin, anti-Th/To, anti-RNA polymerase I and III RNPC3, RuvBL1 and RuvsBL2 (RuvBL1/2), eukaryotic initiation factor 2B (eIF2B), bicaudal D homolog 2 (BICD2)
II. Biomarkers in immune system and the pathomechanism	
II/1 Cytokines	Interleukin-α (IL-α), IL-β and IL-13, IL-18-binding protein isoform (IL18BPa), IL-33, IL-13, IL-4, IL-6, IL-10, IL-1, IL-17A, IL-17B, IL-17E, IL-12, IL-F, transforming growth factor-β (TGFβ), connective tissue growth factor (CTGF)
II/2 Chemokines	Chemokine-ligand 4 (CXCL4), CXCL10, CX3CL1, CCL2
II/3. Vasculopathy	
II/3.1. Early phase of SSc	IL-6, IL-4, IL-13, TGF-β, monocytes, macrophages, CXCL4, platelet-derived growth factor (PDGF), fibronectin, Serpine1, intercellular adhesion molecule 1 (ICAM-1). B-cell activating factor (BAFF), interferon-γ (IFN-γ), CXCL10, CXCL8, angiopoietin 1 and 2 (Ang-1 and 2), angiostatin, resistin, visfatin, C–C motif chemokine ligand 21(CCL21), CXCL11, Semaphorin-3E (Seam3E), IL-35
II/3.2. Active phase of Ssc	TGF-β, VEGF, endoglin, endothelin-1, IL1-α, IL-6, soluble oncostatin M receptor (sOSMR), IL-17F, IL-17E, CXCL5, CX3CL1, resistin, galectin 1, galectin 3, vaspin, chemerin, IL-33, stimulating growth factor (ST2), CXCL4
	Superoxide anion (O• −), hydroxyl radical (OH•), Hydrogen peroxide (H2O2), (HIF-1α and ß), VEGF, fibronectin-1, thrombospondin-1, Proα 2(1) collagen (COL1A2), connective tissue growth factor (CTGF), TGF-ß induced protein (TGF-ßi)
II/3.3 Late phase or fibrotic biomarkers	TGF-β, PDGF, type I and III collagen, YKL-40, CTGF, CXCL5
II/4. Metabolic properties	Adiponectin, leptins, resistin, galectin 1, galectin 3, vaspin, chemerin
II/5. Circulating neurovascular guidance molecules	Ephrins, netrins, slits, semaphorin (Sema3s), Sema3C, nonribosomal peptides (NRPs), slit family (Slit1, Slit2, Slit3), member of the sirtuin family as SIRT1 and SIRT3
III. Biomarkers in the organ manifestation or disease activity	C-reactive protein (CRP), KL-6, vascular cell adhesion molecule (VCAM-1), E-selectin, P-selectin, type III collagen
III/1. ILD or lung	CRP, CTGF, GDF-15, Il-6, CX3CL1, ICAM-1, Von Willebrand factor, Kl-6, surfactant protein (SP-D), CCL18, matrix metalloproteinas 7 (MMP-7), sCD163, CA 15–3, pulmonary surfactant A and D, YKL-40
III/2. PAH and cardiovascular system	NT-proBNP, endothelin-1 and the A-type anti-endothelin (anti-ETaR) receptor, anti-AT1R, anti-centromer antibody, anti-p4,2, CD144 + EMP cadherin, ratio of Cu/Se and ceruloplasmin/SELENOP, midkine and follistatin-like 3 (FSTL3), miRNAlet-7d, blood viscosity level, VEGF, growth differentiating factor 15 (GDF-15), CXCL4, endostatin, endoglin, Von Willebrand factor, sCD163, IL-13, IL-4. IL-10, IL-6. IL1-β, IL13, IL-32, MIF, CCL20, CCL21, CCL23, CXCL16, GDF15, leptin, resistin, adipsin, chemerin, visfatin, interferon- γ (IFN-γ)
III/3. Skin fibrotic markers	Modified Rodnan skin score, thrombospondin 1 (THBS1), cartilage oligomeric matrix protein (COMP), sialic acid binding Ig like lectin 1 (SIGLEC1), interferon induced protein 44 (IFI44), HOXA distal transcript antisense RNA (HOTTIP), SPRY4-IT1, heat-shock-protein (Hsp27), agalactosyl IgG (IgG-Gal), IL-16, adiponectin, terminal differentiation-induced non-coding RNA (TINCR), membrane spanning 4-domains A4A (MS4A4A), GDF-15, BAFF
III/4. Renal involvement	G-patch domain containing 2 like (GPATCH2L), CTNND2, ICAM-1 and VCAM-1, Anti-RNA polymerase III antibodies (anti-RNAP III), complement C3b (C3b), chemerin, E-selectin
III/5. Gastrointestinal involvement	Antibody againts muscarinic-3 (M3R), calprotectin (F-cal), claudin-3, and lipopolysaccharides (LPS)
III/6. Biomarkers of paraneoplastic SSc	Transcription complex RNA polymerase III (Anti-POLR3), anti-NOR90, 2-hydroxyglutarate (2-HG), α-ketoglutaric acid (α-KG)

Our search strategies were designed to identify the best available systematic reviews and relevant literature. However, we have constructed aim, as focusing primarily on the literature in the theme of SSc by pilot key word as “biomarkers” in the last 10 years. Although, after initial scoping, searches carried out the results, and more keywords and synonyms have gathered our development of search strategy. Although, later we restricted some terms to title only, i.e., the “biomarkers in systemic sclerosis” search term and its synonyms. We have selected, almost 30 international publication—peer reviewed original articles and reviews written in English. Searches were applied between February 2019 and January 2024. We have selected the most relevant publications and systematic literature reviews in the aforementioned time-range. This review search strategy was carried out from Google, Google Scholar, and PubMed. By using this itemized strategy, we have found the major appropriate papers and scientific results for this review on the biomarkers in SSc [38].

### The diversity of biomarkers

**Fig. 1:**
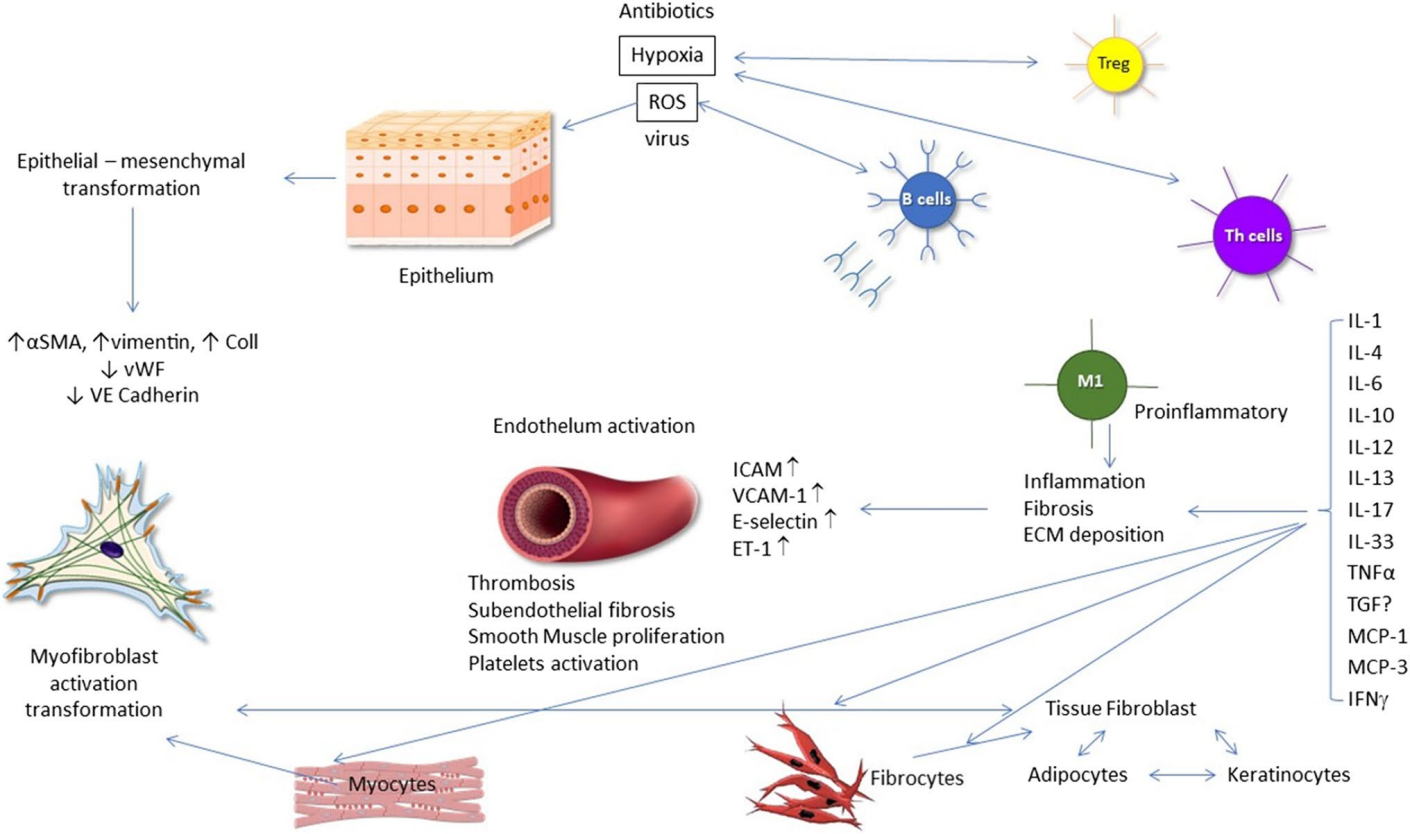
A brief overview of the pathomechanism of systemic sclerosis. (ɑSMA, alpha-smooth muscle actin; Col1, collagen type I; ET-1, endothelin-1; ECM, extracellular matrix; ICAM, intercellular adhesion molecules; IL, interleukin; ROS, reactive oxygen species; TNFɑ, tumor-necrosis alpha; TGFß, transforming growth factor beta; MCP, monocyte chemoattractant protein-1; IFN, interferon; Treg, regulatory T cells; VCAM, vascular cell adhesion molecule-1; VE, vascular endothelial; vWF, von Willebrand factor)

The diversity of biomarkers in systemic sclerosis is a continuously expanding field to monitor the pathomechanism, clinical course, and therapeutic approaches. The biomarkers, as non-invasive and sensitive indicators reflect the physiological and pathological processes, disease prognosis, and the response to therapy. In detail, specific biomarkers are needed for classification, early diagnosis, distinguishing between the subtypes of the disease (lSSc and dSSc), the co-existence of the organ manifestations with the subtypes, clinical course, and the prognosis, as well as for evaluating the therapeutic response [39]. In systemic sclerosis, sensitive and specific, validated biomarkers are not confirmed yet, despite of the overwhelmed and extensive research, except for the NT-pro-brain natriuretic peptide (NT-proBNP) in pulmonary arterial hypertension, the anti-topoisomerase (anti-Scl70) in dSSc, and the anti-centromere antibody in lSSc. However, ANA positivity is one of the criteria in the early onset systemic sclerosis [40]. The modified Rodnan skin score (mRSS) is a functional biomarker and gold standard to measure the disease extension and activity and, however, has numerous difficulties to precisely assess skin involvement. To differentiate the fibrotic skin from the borderline changes or the edema in the early phase is problematic by this assessment [23]. The initial and most critical process in SSc pathogenesis is the vascular dysfunction which leads to the development of PAH and renal crisis. The endothelial cell abnormality is demonstrated by elevated von Willebrand factor levels. The presence of adhesion molecules contributes to the development of early fibrosis and correlates with organ manifestations. VEGF is an important molecule for the assessment of disease progression, and its level is significantly high in early SSc, as well as in cases with worsening of the vital capacity (Fig. 1) [41]. Endothelin-1 (ET-1) as a potential vasoconstrictor, stimulates the smooth muscle cells and has also an important role in obliterative vasculopathy and in Raynaud’s phenomenon. ET-1 correlates strongly with levels of von Willebrand factor and adhesion molecules. The elevated plasma levels of endostatin show positive correlation with the presence of mega-capillaries, digital ulcers, and PAH [42–44].

Biomarkers can be sensitive indicators of the development, the state or progression of SSc, as well they can be used to monitor therapeutic efficacy. In summary, the aforementioned biomarkers are affordable and convenient tools in the clinical practice and aid research as well (Tables 1 and 2).

**Table 2:** Treatment options in systemic sclerosis based on Campochiaro C, Allanore Y. An update on targeted therapies in systemic sclerosis based on a systematic review from the last 3 years. [156])

Treatment options
I. Vascular therapy I/1. Vasodilatators Calcium-channel blockers (CCBs) Beta-blockers Silendafil I/2. Prostacyclin analogues Iloprost (synthetic analogue of prostacyclin PGI2) Flolan Beraprost (active prostacyclin analogue) I/3. Vascular remodelling Bosenthan (anti-endothelin-R) Selective serotonin reuptake antagonists ACEI ARBs I/4. Antioxidants Brobucol Vitamin supplements Selenium, copper, cobalt II. Immunomodulatory therapy II/1. Conventional immunomodulatory therapy Methotrexate Cyclophosphamide Mycophenolate mofetil Azathioprine Low-dose corticosteroid II/2 Biological disease-modifying antirheumatic drug (bDMARD) Rituximab (anti CD-20) Tocilizumab (anti-IL-6) Belimumab (anti-BAFF) Inebilizumab (anti-CD19) Romilkimab (IL-4/IL-13) Abatacept (CTLA4-Ig) Rilonacept (anti-IL-1R) II/3 Targeted synthetic disease-modifying antirheumatic drug (tDMARD) Tocaficitinib (JAK/STAT) II/4 Stem cell transplantation III. Antifibrotic therapy Nintedanib (tyrosine kinase inhibitor) Imatatinib ( protein-kinase inhibitor) Riocguat (stimulator of soluble guanylate cyclase) Pirfenidone (transforming growth factor beta-stimulated collagen production, unknown mechanism) Lenabasum (cannabinoid receptor type 2 agonist) Lanifibranor (peroxisome proliferator-activated receptor agonist)

### Biomarkers in the diagnosis

Generally, in the broad spectrum of biomarkers, the disease-specific autoantibodies have an important role in setting the right diagnosis and also are associated with the clinical manifestations and outcome of the disease [10, 12].

### Autoantibodies

To classify the biomarkers for the diagnostic and clinical categorization, for the assessment of endothelial dysfunction, fibrosis, immunological alterations, and organ manifestations are grouped as follows: autoantibodies, growth factors, cytokines, chemokines, and adhesive molecules.

The early diagnosis and identification of systemic sclerosis subtypes provide better outcome in this progressive disease. Anti-nuclear antibody (ANA) positivity, besides the presence of puffy fingers and Raynaud-phenomenon, is highly representative in the early onset systemic sclerosis. Most of the time, in the early phase of the disease, the phenotype of the two subtypes is common [45].

### ANA patterns

ANA patterns (centromere, nucleolar, RNA polymerase III, Scl-70, U3-RNP, Eukaryotic initiation factor 2B (eIF2B), RuvBL1, and RuvsBL2) reflect the development of subtypes and clinical manifestation of Ssc [46–51]. The anti-Scl-70, anti-U3RNP, anti-Th/To, Bicaudal D homolog 2 (BCID2), Th/To (Rpp25/Rpp38), Ro52, eIF2B, anti-U11/U12 autoantibodies, as well as anti-Pm/Scl highly refer to interstitial pulmonary disease (ILD); however, pulmonary arterial hypertension (PAH) appears often in the presence of anti-centromere, anti-U3RNP, anti-Th/To positivity [51–54]. Ssc-myositis overlap syndromes are associated with anti-Ku, anti-RNP and anti-PM/Scl, and RuvBL1 and RuvsBL2 (RuvBL1/2) antibodies [48, 55, 56]. Correlation has been shown between malignancy and RNA-binding region containing three (RNPC3) or RNA polymerase III (RNA pol III) [57, 58] (Table 3).

**Table 3:** ILD-associated biomarkers

Biomarker	Function	Clinical association	Response to the therapy
KL-6 (Krebs von den Lungen-6)	Type II pneumocyte mucinosous glycoprotein	Most informative biomarkers for ILD	Yes [115]
SP-A and SP-D (surfactant protein-A and D)	Produced by type II pneumocyte	Capillary and alveolar barrier distortions	Not known [120, 121]
CCL2, CCL18 (pulmonary and activation-regulated chemokine)	T cell chemotaxis, migration	ILD progression and mortality	Not known [59, 60]
YKL-40 (chitinase-3-like protein 1)	Tissue activator	Worse ILD prognosis and mortality	Not known [118]
Calprotectin	60% soluble protein (neutrophil granulocyte, monocyte, macrophage, epithelial cells)	Gastrointestinal symptoms, ILD, more severe SSc form	Yes [123]
CXCL3 and CXCL4	Cell migration, inflammation	ILD, kidney	Not known [117]
Anti-Ro 52/TRIM21 (tripartite motif-21)	Mononuclear cells ubiquitin ligase	ILD, worse prognosis	Not known [114]
OX40L	Direct effect on MMPs expression, fibrosis	dSSc-ILD, worse prognosis	Not known [62]
MCP-1 (monocyte chemoattractant protein-1)	T cell and monocyte migration, cell adhesion	ILD progression	Not known [119]
Anti-Scl70	Anti-DNA topoisomerase antibody	ILD, FVC worsening	Not known [35, 36]

This manuscript has been supported by TKP 2021-EGA-22

### Biomarkers in the immune system and pathomechanism

The complex and heterogenous pathogenesis of Ssc is characterized by vasculopathy, immune cell, and molecular mediator activation, as well as the accumulation and deposition of fibroblasts. In the genetic predisposition along with exogenous stimuli, the activation of the innate/adaptive immune system regulates the endothelial and fibroblast homeostasis, leading to the sequel of pathogenic processes [10].

### Cytokines

Systemic sclerosis and its manifestations are mostly characterized by fibrosis during the disease duration. The IL1-like cytokines, as IL1α and β, were detected in SSc patients compared to healthy controls, and elevated ILα levels were observed in patients with DU; higher concentrations of ILβ and IL-13 were described in PAH. IL-18-binding protein isoform (IL18BPa) was associated with the pulmonary arterial wedge pressure (sPAP) [59]. IL-33 is correlated with the sPAP, DU, and diastolic dysfunction, as well. Remarkable elevated levels of IL-13, IL-4, IL-6, and IL-10 were detected in patients with PAH and cardiac manifestations [61, 62]. Overall, significant differences of IL-17 have not been observed in SSc patients versus controls; however, IL-17A, IL-17B, IL-17E, and IL-17F were significantly elevated in SSc patients, and IL-17E and IL-F have been associated with DU [60–62].

There is a wide spectrum of biomarkers reflecting fibrotic processes and can aid with the therapeutic approach. TGFβ stimulates the synthesis of extracellular molecules directly and decreases the matrix metalloproteinases. At the same time, TGFβ changes the phenotype of tissue fibroblasts and initiates transformation into myofibroblast. CTGF is also a significant factor for fibrosis; however, it is not clear if TGFβ or CTGF was the better biomarker for fibrosis processes.

The PDGFα and β are also very informative and therapy-sensitive indicators and hinder the efficacy of nintedanib therapy [63–65].

Mononuclear cell infiltration is significant both in the internal organs and skin. The infiltrating phenotypically altered T cell populations release cytokines and growth factors, which usually leads to the development of extensive collagen mass. In systemic sclerosis, the pathogenic role makes IL-6 an excellent target cytokine, as tocilizumab has been proven in ILD, PAH. and musculoskeletal involvements [66]. Otherwise, lower IL-6 levels have been detected in patients with DU [67].

The other prominent pro-inflammatory cytokine, the macrophage migration inhibitory factor (MIF), has been associated with PAH [68]. IL-2 receptor has been shown to be a relevant biomarker of the disease progress and skin severity. TNF-alpha is unquestionably one of the key markers in the pathophysiology of SSc and could reflect the progression of pulmonary disease. However, it has not been clarified whether TNF alpha or its receptor was the more informative biomarker in this disease [69, 70]. Taken together, cytokines are excellent biomarkers reflecting vascular abnormalities and PAH in SSc.

### Chemokines

Chemokines (CXCL4, CXCL10, CX3CL1) have also a significant impact in the progression of SSc. CXCL4 is a prohibitor of IFN-γ and could enhance the skin fibrosis. CXCL10 is predictive in the early onset SSc. Digital ulceration and pulmonary fibrosis are reported to be associated with CX3CL1 through its role in migration and adhesion [71]. Interstitial pulmonary disease and pulmonary arterial hypertension are highly responsible for the mortality and morbidity in SSc. Several molecules are confirmed to reflect ILD severity, and most of them are expected to be potentially useful biomarkers. The endothelial microparticles, e.g., CD144 + plays an important role in cell–cell interactions and signaling. The serum concentration of these molecules is significantly elevated in PAH. The lung-epithelial surfactant proteins are relevant diagnostic markers in ILD. KL-6 shows the fibrosis severity in ILD. ILD severity is associated with CCL-2, CXC4, and PF-4 that are produced by immune cells. CCL-18 has a pivotal role in the collagen synthesis and is a strong prognostic factor in ILD severity [72]. YKL-40 (chitinase-3-like protein 1)—as a tissue activator—is also a very important biomarker of ILD prognosis. The fecal and serum calprotectin—however is not a chemokine—is also a good biomarker both for the gastrointestinal manifestation and ILD. Furthermore, calprotectin is therapy sensitive; therefore, it could be validated for monitoring the symptoms in SSc in the future [72–74].

Chemokine alterations can reflect the pathological pathways, e.g., stable serum CCL-2 level and decreased CXCL-10 level refer to the Th1 shift to Th2 pathway. Anti-Ro52 antibodies are biomarkers of infective pulmonary diseases and predictive for worse outcome in ILD. The OX40-OX40L axis correlates with the extension of fibrosis in the lung and skin, as well [71, 75] (Table 2).

### Circulating neurovascular guidance molecules

Several neural molecules have been shown to regulate vascular remodelling, as ephrins, netrins, slits, and semaphorins. The balance of neurovascular communication is essential in the neurovascular stability. In SSc, the role of secreted class III semaphorin (Sema3s) is related to angiogenesis. Sema3C has both pro- and anti-angiogenic factor functions; Sema3E has been associated with early vascular abnormalities [76].

Increased level of NRPs has been described in Ssc patients with PAH. Regarding the Slit family (Slit1, Slit2, Slit3), a Slit2-SSc association has been depicted in the early onset as a peripheral vascular biomarker. Among the sirtuins (SIRTs), SIRT1 and SIRT3 are decreased in SSc and being related to DU [77, 78].

### Metabolic properties

Adiponectin is a bifunctional hormone as having pro-and anti-inflammatory roles in different diseases. In SSc, decreased adiponectin concentrations have been found significantly increasing concentration levels which have been shown after prostaglandin analogue treatment [79]. Leptins activate the pro-inflammatory cytokines and enhance angiogenesis. However, some studies have not reported significant differences in serum leptin levels between SSc patients and controls, while others have shown increased level of leptin in SSc patients with PAH [80]. Similarly, resistin levels did not differ between the two groups; however, increased level of resistin was detected in patients with DU and PAH. Galectin 1 is associated with telangiectasias; galectin 3 refers to the development of DU [80, 81]. On the contrary, the level of vaspin was decreased in SSc patients with DU [82]. Chemerin has pro- and anti-inflammatory effects, depending on the circulating immune cells and micro-environmental background. Chemerin was significantly increased in Ssc-PAH, as well [83, 84].

### Biomarkers in the early phase of Ssc

In the early phase of Ssc, the vascular dysfunction is presented by the aberrant cell–cell interaction by increased expression of adhesion molecules, such as VCAM-1, ICAM, E-selectin, and the growth factors as TGFβ, endothelin-1 (ET-1), and PDGF [85]. The permanent vasoconstriction is strongly triggered by the ET-1, angiotensin, and activation of leucocytes. The activation of thrombocytes also contributes to the vasoconstrictions and vWFAg; thrombospondin and thrombomodulin are also possible biomarkers in the early phase of Ssc [86]. On the contrary, the aberrant vasodilatation can be detected by lower concentrations of NO as well as the lower expression of NO3 gene [87]. Furthermore, the imbalance of angiostatic factors, such as angiostatin, endostatin, chemokin ligand 4 (CXCL4), thrombospondin, (IL-4) and the angiogenic factors, VEGF, PDGF, TGF-β1, PGF-2, PIGF, ET-1, MCP-1, TNF-α, IL-8, E-selectin, P-selectin, and urokinase plasminogen activator receptors reflect the development of abnormal density capillaries and angiogenesis [87, 88].

### The biomarkers in the active and late phases of SSc

In the active and late phases, a broad spectrum of biomarkers can reflect various complications of the disease and predict the progression of the tissue injury.

### Biomarkers of oxidative stress

#### The reactive oxidative species (ROS)

Reactive oxidative species (ROS) may have a great impact in the pathogenesis of Ssc, including their effect on endothelial dysfunction, fibrosis development, the innate and adaptive immune system, and the development of the autoantibodies [89]. Vasculopathy, the hallmark feature of Ssc, is signified by the perivascular mononuclear infiltration, endothelial injuries, and vascular and extracellular matrix remodelling—alteration of the vessels and capillary structure and functions. The extensive flow of reactive oxygen species, such as superoxide anion (O•‾), hydroxyl radical (OH•), and hydrogen peroxide (H2O2) produced by endothelial cells, smooth muscle cells, and fibroblasts, is responsible for the vascular abnormalities [90, 91]. The Ssc-specific vascular manifestations—the inverse reaction of capillaries—are detectable in the early phase of Ssc, and later ischaemic ulceration may be resulted by the dysregulated ROS milieu. The contractile and relaxation function of the vascular smooth muscle cells is also affected by both the ROS and the increased expression of ROS-induced contractile proteins. Besides the vasoconstriction effects, the elements of the ROS, e.g., overproduction of superoxide and H2O2 may drive vasodilatation resulting in the biphasic response in Ssc [92].

The previous factors and other reactive signaling molecules as NO• and hydrogen sulfide (H2S) altogether interact and cause vascular dysregulation [93, 94].

#### The molecular response biomarkers of hypoxia

Severe hypoxia is the most potent pathogenic risk for the vascular abnormalities. There are several molecular responses to hypoxia including the expression of hypoxia-inducible factor-1 (HIF-1α and ß) and dysregulated cytokines exposure. Hypoxia leads to reduced capillar density, impaired vascular permeability, and diffusion. The upregulation of the extracellular matrix proteins and altered function of vessels are catalyzed by hypoxia [86, 95]. In Ssc, decreased HIF-1α protein levels have been measured despite the severe hypoxia [96]. This paradox mechanism could be defined by HIFα -independent pathways. The VEGF-dependent angiogenesis induced by HIF-1 could be also a possible key point in the hypoxia-induced angiogenesis and vasculopathy. VEGF could be a prominent biomarker in the chronic vascular process of Ssc as both the VEGF levels and the VEGF receptor 1 and 2 are overexpressed as well cause tissue damage. VEGF could also induce hypoxia and malnutrition, and hypoxia could maintain the upregulation VEGF, vice versa [97, 98]. In the chronic, fibrotic lesions, there are several other factors have been described, such as fibronectin-1, thrombospondin-1, proα 2(1) collagen (COL1A2), connective tissue growth factor (CTGF), and TGF-ß induced protein (TGF-ßi) [99, 100].

#### The anti-oxidative enzymes and its cascade

The normal differentiation and activation of B and T cells are catalyzed by anti-oxidative enzymes, such as Gpx1 and catalase. Oxidative stress can lead to the increased inflammatory cascade and IL-4, IL-13 production. Similarly, to other autoimmune disorders, Th-17 levels are also increased, and the production of the Treg cells are decreased in Ssc. These observations underline the positive effects of the anti-oxidant or anti-stress therapy in the inflammatory and autoimmune process in Ssc [101, 102]. The activity of NLRP3 is reduced by H2O2 scavanger catalase and could contribute to the fibrosis development. Moreover, in endothelial cells, NLRP3 activation is triggered by oxidative stress [103, 104]. The “M2-type” macrophages—as subtype of the macrophage/monocyte—polarization are strongly affected by oxidative stress through signal transducer and activator of transcription 6 (STAT6) induction [105]. The pro-fibrotic cytokines, such as TGF-ß and IL-1ß, are highly important in the fibrotic processes and are able to stimulate the elements of ROS, while increased expression of ROS triggers the fibroblast activation to express these cytokines, as well [106]. Members of the metallo-proteinases (MMPs) are also strongly linked to fibrotic processes, the pulmonary arterial hypertension, and the skin and pulmonary fibrosis. MMP-9, MMP-12, and MMP13 levels can be potential biomarkers to monitor the activity of the ROS [107–109].

Development of autoantibodies and the activation of ROS can also be associated. The H2O2-induced protein oxidation can lead to changes of the epitopes and trigger autoantibody production. On the other hand, the antioxidant system or enzymes are targeted by the autoantibodes, as anti-peroxiredoxin and anti-methonine sulfoxide reductase (MSRA) maintain the oxidative stress in Ssc [110–112].

Finally, oxidative stress contributes to tissue damage and the internal and skin fibrosis by increased amino acid and protein hydroperoxide (HP) levels in Ssc. Elevated eosinophilia has been shown in the skin ulcers, elevated CRP levels, cellular fibronectin, and mild anemia along with HP. Therefore, fibronectin, eosinophil cell counts, and hemoglobin levels also could be potent biomarkers for disease activity [113].

### Other vascular biomarkers

The vascular biomarkers are presented in very early Ssc, as microangiopathy can appear rapidly. The small vessel damage and chronic hypoxia could be intensified by angiogenic and fibroproliferative factors, also. Antibodies against interferon-inducible protein 16 refer to digital ischemia [114]. Endostatin is associated with giant capillarity abnormalities and clearly appears at the onset of right ventricular systolic pressure [115]. Endoglin has a remarkable role in angiogenesis, and its level is significantly elevated in patients with DU, associated with anti-centromer antibodies, ILD, and PAH. The endoglin correlates positively with telangiactasia especially hereditary hemorrhagic telangiactasia. Von Willebrand factor (vWF) and ADAMTS-13 are also a positive biomarkers for disease activity and severity in ILD and PAH [116, 117].

### Markers of pulmonary hypertension (PAH) and ILD

A subset of Ssc patients with pulmonary artery hypertension and pulmonary fibrosis, reflecting interstitial lung disease, have the worst clinical outcome. These two progressive phenotypes of the disease represent the leading morbidity and mortality in Ssc.

#### The diagnostic biomarkers of PAH

Right heart catheterization (RHC) is essential for the diagnosis of pulmonary hypertension (PAH) in SSc, also. Although, RHC is an invasive method, it is suggested to use this procedure in cases of high-risk patients [118]. Validated non-invasive and sensitive biomarkers are essential for detecting PAH. The NT-proBNP is a sensitive but not specific marker for PAH in SSc as elevated NT-proBNP level is also associated with left ventricle dysfunction and renal insufficiency. NT-proBNP is correlated with the skin fibrosis, and its level is higher in dSSc [119, 120]. Two important biomarkers, as endothelin-1 and the A-type anti-endothelin (anti-ETaR) receptor antibody are representative for PAH, ILD, and DU. Both markers reflect sensitively for bosentan. The anti-receptor antibody (anti-AT1R) is elevated in decreased DLCO and PAH. The anti-centromer antibody, anti-p4,2, and CD144 + EMP cadherin have a strong correlation with the DLCO < 70 and PAH [121, 122]. FSTL3 expression is stimulated by heart failure and contributes to the activation of fibroblasts leading to increased cells adhesion and collagen synthesis [123]. The human lethal-7 (let-7-d) is another promising biomarker in PAH [124]. Selenium has a potential role in the oxidative stress therefore the elevated Cu/Se rate is important in patients with PAH and fibrosis, also [125].

#### The diagnostic process of ILD

ILD and PAH, as cardiopulmonary manifestations of SSc, are the two major causes of morbidity and mortality in SSc [126]. The mortality in patients with PAH and/or ILD is significantly higher with these comorbidities. Scleroderma renal crisis—as characterized by hypertension and renal failure—is a life-threating condition; however, its prevalence declined after the preferable introduction of angiotensin convertase inhibitor (ACE) [127]. The progressive phenotype of ILD could be identified and followed by forced expiratory volume (FEV1), forced vital capacity (FVC), and DLCO. High-resolution computed tomography (HRCT) is frequently used to clarify and detect the patterns of the pulmonary involvement [128]; however, we must take into consideration the frequented radiation exposure of the HRCT. Recently, the importance and role of the biomarkers in ILD/PAH is more emphasized in the clinical practice, as well [129].

#### The chemokines and other biomarkers of ILD

In BALF, behind the autoantibodies (anti-Scl-70, anti-centromer antibodies, anti-Ro52), CCL18, macrophage 2-derived protein, has been also described to be sensitive for monitoring the progression of SSc-ILD. KL-6 (Krebs von den Lungen-6), MMP7, and MMP12 are good prognostic factors in the early lung involvement or Ssc-ILD, overall [130–132]. CCL2 is related to ILD progression and poor prognosis. Some proteome-wide studies have shown that CXCL3 and CXCL4 levels were significantly higher in SSc-ILD patients, otherwise did not correlate with the severity of the disease [133]. Dichev et al. described the regulation of serum 40-kDa heparin-and-chitin binding glycoprotein (YKL-40) and plasma miR-214 levels and found that both biomarkers could distinguish between patients with SSc, dcSSc, and lcSSc [134]. The serum monocyte chemoattractant protein-1 (MCP-1) levels in the BAL are known to be a good marker to be correlated with the clinical course of ILD patients and could predict the clinical course of ILD [135]. SP-A and SP-D are elevated in patients with Ssc and correlate with decreased DLCO. SP-D was detected as could show the state of pulmonary fibrosis but did not follow the progression of the pulmonary fibrosis progression [136, 137]. Soluble OX40L also correlates with the worsening of lung and skin fibrosis. OX40L has a profibtotic effect and triggers the influx of the inflammatory cells into tissues leading to fibrosis [138]. Beyond, the proven role of calprotectin in Ssc patient with GE manifestation, calprotectin is also a promising marker in BALF connected with inflammatory pulmonary fibrosis [74] [Table 4].

**Table 4:** Systemic sclerosis-specific antibodies

Biomarker	Classification	Clinical association
Anti-Scl-70	Anti-DNA topoisomerase antibody	Diffuse cutan SSc, pulmonary fibrosis [35, 36]
anti-CENP-A (anti-centromere Ab (ACA))	Anti-kinetochore protein antibody	Limited cutan SSc, arterial pulmonary hypertension (10–20%) [35, 36]
Anti-Pm-Scl	110–120 kDA nuclear and nucleolar protein antibody	PM/SSc overlap [45]
Antifibrillarin	Az U3-RNP 34 kDa nuclear protein component antibody	Diffuse cutan Ssc [35]
Anti-Th/To	RNAase P ribonucleoprotein antibody	Limited cutan SSc, pericarditis, ILD [35]
Anti-RNA polymerase I and III RNPC3	RNA polymerase antibody RNA binding region containing 3 antibodies	Diffuse cutan SSc, renal involvement, malignancy [46, 47] Malignancy, ILD, GI dysmotility, myopathy [48]
RuvBL1 and RuvsBL2 (RuvBL1/2)	ATP-binding protein belonging to the AAA + (ATPase associated with diverse cellular activities) superfamily of ATPases	Diffuse cutaneous disease, inflammatory myositis overlap [40]
Eukaryotic initiation factor 2B (eIF2B)	Cytoplasmic multimeric protein consisting of 5 subunits	Diffuse cutaneous disease, ILD [39]
Bicaudal D homolog 2 (BICD2)	94 kDa protein and one of two human homologs of Drosophila bicaudal-D	Inflammatory myositis, ILD [45]

### Skin fibrosis markers

Besides the modified Rodnan skin score, further non-invasive but more objective biomarkers are needed to evaluate the skin involvement in SSc. The heat-shock protein, as a pro-inflammatory molecule, is increased in dSSc than in lSSc or healthy individuals [139]. IgG-Gal and IL-16 cytokine show a positive correlation with mRSS and skin severity, and subtypes of SSc can be assessed by this molecule [140]. Inverse correlation has been established between the adiponectin and skin fibrosis or mRSS [141]. The genetic analysis of the scleroderma skin has a promising candidate biomarker pattern. The THBS1, COMP, SIGLEC1, and IFI44 are correlated moderately with the mRSS, and further analyses have confirmed that HOTTIP and SPRY4-IT1 show positive correlation with mRSS; otherwise, ANCR and SPRY4-IT1 are significant biomarkers for PAH [142].

### Potential renal biomarkers

The renal manifestation is commonly appearing in SSc patients. The scleroderma renal crisis (RSC) could be a life-threatening episode in SSc. The exact role of anti-RNS polymerase III antibody is unknown. The pathogenic role of GPATCH2L, CTNND2, ICAM-1, and VCAM-1 is confirmed [143]. Additionally, there are some other molecules, such as C3b deposits and chemerin are depicted to be relevant biomarkers in several autoimmune disorders and in SSc, as well [144].

### Gastrointestinal biomarkers

Calprotectin levels are highly sensitive but not specific biomarker of GI manifestation [145]. The antibody againts Muscarinic-3 (M3R) receptor and RNA binding region containing 3 has been detected in Ssc with GI dysmotility [146, 147]. GI manifestations could be the early onset in Ssc, and the calprotectin (F-cal) is described to be presented in the early phase of the disease as well. However, F-cal has not shown associations with the esophageal radiological alterations. Testing of the calprotectin at the time of the diagnosis or suspicion of Ssc onset can be useful [148]. In another cross-sectional study, Stec et al. have found that among the serum intestinal permeability markers as intestinal fatty acid binding protein, claudin-3 and lipopolysaccharides (LPS) were markedly different and elevated in Ssc patients with GI abnormalities. Higher levels of LPS and claudin-3 were associated with a shorter duration of the disease. Moreover, in this group, the LPS concentrations were related to ILD. Concomitant esophageal dysmotility was associated with a decrease in LPS in patients with SSc. Both calprotectin and LPS are established as early biomarkers in gastrointestinal malformations [37].

### Biomarkers of paraneoplastic SSc

Individuals with systemic sclerosis have a significantly higher risk for developing cancer. Although, the development of cancers in SSc are associated with the presence of autoantibodies and several provoking and genetic factors [149]. Chronic inflammation, tissue damage, and immune-suppressive agents heightened the link between cancer development and Ssc [150]. On the other hand, SSc could appear as a paraneoplastic syndrome, as cancer-induced autoimmunity [151]. Onishy et al. have found an increased tendency of hematological, lung, liver, and bladder cancer in females and non-melanomatous cancer in males. Anti-POLR3 positive patients with diffuse scleroderma have a higher risk for breast, prostate, and tongue cancer [152]. Paraneoplastic syndrome manifestations and SSc development can happen simultaneously. The anti-NOR90 antibody is reported in lSSc and in myelodysplastic syndromes. In anti-NOR90-positive patients, IDH1 mutation causing elevated 2-hydroxyglutarate (2-HG) levels and concomitant α-Ketoglutarate octyl ester (α-KG), dimethyl- α-KG inhibition, and elevated TGFβ levels and myofibroblast migration [153].

## Recommendation for clinicians

Beyond the availability of on the numerous biomarkers we have summarized in details, there is a critical step to further evaluate their clinical implementations. Although the clinical utility of all biomarkers has been assessed in the last decades, it still remains difficult to rank the clinical usefulness of these molecules [153]. The predictive values of each biomarker could be significant; therefore, we strongly believe that several biomarkers should be used simultaneously to predict, monitor, or guide the treatment of SSc.

Although there is a great variety of biomarkers in the SSc pathogenesis, clinical course prognosis, and response to therapy, however, only some essential biomarkers are available in the clinical practice as prognostic tools for clinicians to focus on the early onset of SSc through the disease duration, which indicate the most appropriate treatment or failure the therapy [154].

Firstly, the presence of autoantibodies predicts and confirms the onset of the disease along with the clinical symptoms; therefore, the ANA patterns assist to evaluate the subtypes and the main clinical manifestations of Ssc. Moreover, there are some autoantibodies which are important to be highlighted in overlap syndromes (e.g., anti Pm/Scl 70).

Secondly, the follow-up and management of Ssc are required by multidiscipline approach. For cardiologist and pulmonologists, the vascular biomarkers are useful to predict the severity and onset of the PAH and ILD. Otherwise, the right heart catheterization (RHC) with the NT-proBNP is essential routine diagnostic procedure for the diagnosis of pulmonary hypertension (PAH), ILD, and PU. KL-6 and pulmonary surfactants A and D (PS-A, -D) are also key proteins and have a positive correlation with the pulmonary fibrosis.

The pulmonary status should be followed by forced expiratory volume (FEV1), forced vital capacity (FVC), and DLCO. High-resolution computed tomography (HRCT) is one of the most frequently useful tools to detect the patterns of the pulmonary involvement [112]. In BALF, certain autoantibodies and molecules have been also described to be sensitive for monitoring the progression of SSc-ILD.

Calprotectin levels are highly sensitive but not specific biomarker of GI manifestation and idiopathic pulmonary fibrosis.

Selenium, as a trace element nutrient and antioxidant enzyme, the Cu/Se rate is a practicable factor to predict and follow PAH and fibrosis, as well. Sclerodermal renal crisis and the high risk for cancer are associated with the presence of autoantibodies and several provoking and genetic factors; although the exact predictive biomarkers are not avaiable in the clinical routine, therefore, the regular follow-up of blood pressure, renal function, is essential. Also, the rapid progression, the late onset of the disease, can indicate parenoplastic syndrome.

There is controversial evidence of biomarkers in the current clinical practice; therefore, it is pivotal that research should be conducted to continuously evaluate “biomarker patterns” and to aid clinicians to use them in the daily clinical care [153, 154].

## Conclusions

In systemic sclerosis, the importance of biomarkers is pivotal in the differential diagnosis, for classification to subgroups, to decipher manifestations, to assess disease activity, to monitor prognosis, response to therapy, and to establish personalized therapy, as well. Despite of the general scientific knowledge of the pathomechanism, breakthrough treatment options are still lacking. Only in pulmonary arterial hypertension where the molecular pathomechanism is better known, targeted therapy has been shown to slow down disease progression. However, well-defined or “clear” biomarkers to predict the prognosis have not been validated, yet. Strong biomarkers are needed to distinct the early and late phases of Ssc, as well as the vascular and fibrotic processes [155]. Unfortunately, the specificity and sensitivity of current biomarkers are variable. Finally, validated, “cost–benefit” biomarkers as well as a set of biomarkers, and biomarker-patterns to monitor response to the therapy are essential. As of today, individually tailored biomarkers are not available, as their sensitivity and specificity can differ from patient-to-patient. Several potential biomarkers for the prognosis, vascular injuries, fibroproliferative processes, and organ damages are under evaluation [156–158].

Further efforts for the evaluation of biomarker patterns are pivotal from basic research and clinical science centers in order to optimize patient follow-up and clinical care.

## References

[1] Rosendahl A, Schönborn K, Krieg T (2022) Pathophysiology of systemic sclerosis (scleroderma). Kaohsiung J Med Sci 38:187–195. 10.1002/kjm2.12505 35234358 10.1002/kjm2.12505 PMC11896191

[2] Keret S, Rimar D, Lansiaux P et al (2023) Differentially expressed genes in systemic sclerosis: towards predictive medicine with new molecular tools for clinicians. Autoimmun Rev 22:103314. 10.1016/j.autrev.2023.213324 36918090 10.1016/j.autrev.2023.103314

[3] Gourh P, Safran SA, Alexander T et al (2020) HLA and autoantibodies define scleroderma subtypes and risk in African and European Americans and suggest a role for molecular mimicry. Proc Natl Acad Sci 117:552–562. 10.1073/pnas.1906593116 31871193 10.1073/pnas.1906593116 PMC6955366

[4] Abignano G, Hermes H, Piera-Velazquez S et al (2021) Global gene expression analysis of systemic sclerosis myofibroblasts demonstrates a marked increase in the expression of multiple NBPF genes. Sci Rep 11:20435. 10.1038/s41598-021-99292-y 34650102 10.1038/s41598-021-99292-y PMC8516909

[5] Stern EP, Denton CP (2015) The pathogenesis of systemic sclerosis. Rheum Dis Clin North Am 41:367–382. 10.1016/j.rdc.2015.04.002 26210124 10.1016/j.rdc.2015.04.002

[6] Kerick M, González-Serna D, Carnero-Montoro E et al (2021) Expression quantitative trait locus analysis in systemic sclerosis identifies new candidate genes associated with multiple aspects of disease pathology. Arthritis Rheumatol 73:1288–1300. 10.1002/art.41657 33455083 10.1002/art.41657

[7] Zhou X, Tan FK, Wang N et al (2003) Genome-wide association study for regions of systemic sclerosis susceptibility in a Choctaw Indian population with high disease prevalence. Arthritis Rheum 48:2585–2592. 10.1002/art.11220 13130478 10.1002/art.11220

[8] Wasson CW, Abignano G, Hermes H et al (2020) Long non-coding RNA HOTAIR drives EZH2-dependent myofibroblast activation in systemic sclerosis through miRNA 34a-dependent activation of NOTCH. Ann Rheum Dis 79:507–517. 10.1136/annrheumdis-2019-216542 32041748 10.1136/annrheumdis-2019-216542 PMC7147169

[9] Fearon AE, Slabber CF, Kuklin A et al (2021) Fibroblast growth factor receptor 3 in hepatocytes protects from toxin-induced liver injury and fibrosis. iScience 24:103143. 10.1016/j.isci.2021.103143 34646985 10.1016/j.isci.2021.103143 PMC8497853

[10] Cutulo M, Soldano S, Smith S (2019) Pathophysiology of systemic sclerosis: current understanding and newinsight. Expert Rev Clin Immunol 15:753–764. 10.1080/1744666X.2019.1614915 31046487 10.1080/1744666X.2019.1614915

[11] Chizzolini C, Boin F (2015) The role of the acquired immune response in systemic sclerosis. Semin Immunopathol 37:519–528. 10.1007/s00281-015-0509-1 26152639 10.1007/s00281-015-0509-1

[12] Asano Y (2020) The pathogenesis of systemic sclerosis: an understanding based on common pathologic cascade across multiple organs and additional organ-specific pathologies. J Clin Med 9:2687. 10.3390/jcm9092687 32825112 10.3390/jcm9092687 PMC7565034

[13] Bellando-Randone S, Matucci-Cerinic M (2019) Very early systemic sclerosis. Best Pract Res Clin Rheumatol 33:101428. 10.1016/j.berh.2019.101428 31810547 10.1016/j.berh.2019.101428

[14] Frantz C, Auffray C, Avouac J (2018) Regulatory T cells in systemic sclerosis. Font Immunol 15(9):2356. 10.3389/fimmu.2018.02356 10.3389/fimmu.2018.02356 PMC6196252 30374354

[15] Sakkas LI, Katsiari CG, Daoussis D, Bogdanos DP (2023) The role of B cells in the pathogenesis of systemic sclerosis: an update. Rheumatology 62:1780–1786. 10.1093/rheumatology/keac578 36218415 10.1093/rheumatology/keac578

[16] Negrini S, Fenoglio D, Parodi A et al (2017) Phenotypic alterations involved in CD8+ treg impairment in systemic sclerosis. Front Immunol 8:18. 10.3389/fimmu.2017.00018 28154567 10.3389/fimmu.2017.00018 PMC5243838

[17] Ah Kioon MD, Tripodo C, Fernandez D et al (2018) Plasmacytoid dendritic cells promote systemic sclerosis with a key role for TLR8. Sci Transl Med 10:eaam845. 10.1126/scitranslmed.aam8458 10.1126/scitranslmed.aam8458 PMC9865429 29321259

[18] Khalil N, Bereznay O, Sporn M, Greenberg AH (1989) Macrophage production of transforming growth factor β and fibroblast collagen synthesis in chronic pulmonary inflammation. J Exp Med 170:737–737. 10.1084/jem.170.3.727 10.1084/jem.170.3.727 PMC2189427 2475572

[19] Trombetta AC, Soldano S, Contini P et al (2018) A circulating cell population showing both M1 and M2 monocyte/macrophage surface markers characterizes systemic sclerosis patients with lung involvement. Respir Res 19:186. 10.1186/s12931-018-0891-z 30249259 10.1186/s12931-018-0891-z PMC6154930

[20] Rech TF, Moraes SBC, Bredemeier M, et al (2016) Matrix metalloproteinase gene polymorphisms and susceptibility to systemic sclerosis. Genet Mol Res 15. 10.4238/gmr15049077. 10.4238/gmr15049077 28002595

[21] Asano Y, Sato S (2015) Vasculopathy in scleroderma. Semin Immunopathol 37:489–500. 10.1007/s00281-015-0505-5 26152638 10.1007/s00281-015-0505-5

[22] Hinz B, Phan SH, Thannickal VJ et al (2007) The myofibroblast: one function, multiple origins. Am J Pathol 170:1807–1816. 10.2353/ajpath.2007.070112 17525249 10.2353/ajpath.2007.070112 PMC1899462

[23] Khanna D, Furst DE, Clements PJ et al (2017) Standardization of the modified Rodnan skin score for use in clinical trials of systemic sclerosis. J Scleroderma Relat Disord 2:11–18. 10.5301/jsrd.5000231 28516167 10.5301/jsrd.5000231 PMC5431585

[24] Jerjen R, Nikpour M, Krieg T et al (2022) Systemic sclerosis in adults. Part I: Clinical features and pathogenesis. J Am Acad Dermatol 87:937–954. 10.1016/j.jaad.2021.10.065 35131402 10.1016/j.jaad.2021.10.065

[25] Riccardi A, Martinroche G, Contin-Bordes C et al (2022) Erosive arthritis autoantibodies in systemic sclerosis. Semin Arthritis Rheum 52:151947. 10.1016/j.semarthrit.2021.11.013 35000789 10.1016/j.semarthrit.2021.11.013

[26] Sakchaikul A, Chowchuen P, Foocharoen C, Thammaroj P (2021) Prevalence and clinical association with acro-osteolysis in early systemic sclerosis. Clin Exp Rheumatol 39:1093–1098. 10.55563/clinexprheumatol/vggbdq 33427611 10.55563/clinexprheumatol/vggbdq

[27] Ostojic P, Knezevic-Apostolski S, Djurovic N et al (2021) Neurological and electroneurography findings in patients with systemic sclerosis and symptoms of neuropathic pain in extremities. Acta Neurol Belg 121:205–209. 10.1007/s13760-018-1048-z 30465254 10.1007/s13760-018-1048-z

[28] Smith V, Ickinger C, Hysa E et al (2023) Naifold capillaroscopy. Best Pact Res Clin Rheumatol 37:101849. 10.1016/j.berth.2023.101849 10.1016/j.berh.2023.101849 37419757

[29] Muktabhant C, Thammaroj P, Chowchuen P, Foocharoen C (2021) Prevalence and clinical association with calcinosis cutis in early systemic sclerosis. Mod Rheumatol 31:1113–1119. 10.1080/14397595.2021.1886654 33566708 10.1080/14397595.2021.1886654

[30] Chatterjee S, Prayson RA (2020) Concurrent anti-PM-Scl antibody-associated systemic sclerosis and inclusion body myositis—report of two cases and review of the literature. Semin Arthritis Rheum 50:498–502. 10.1016/j.semarthrit.2019.11.008 31806155 10.1016/j.semarthrit.2019.11.008

[31] Muangchan C, Harding S, Khimdas S et al (2012) Association of C-reactive protein with high disease activity in systemic sclerosis: results from the Canadian Scleroderma Research Group. Arthritis Care Res 64:1405–1414. 10.1002/acr.21716 10.1002/acr.21716 22556030

[32] Young A, Moles VM, Jaafar S et al (2021) Performance of the DETECT algorithm for pulmonary hypertension screening in a systemic sclerosis cohort. Arthritis Rheumatol 73:1731–1737. 10.1002/art.41732 33760392 10.1002/art.41732 PMC8403104

[33] Meyer KC, Raghu G, Baughman RP et al (2012) An official American Thoracic Society clinical practice guideline: the clinical utility of bronchoalveolar lavage cellular analysis in interstitial lung disease. Am J Respir Crit Care Med 185:1004–1014. 10.1164/rccm.201202-0320ST 22550210 10.1164/rccm.201202-0320ST

[34] Ma L, Zhu Q, Zhang Y et al (2021) Esophagus involvement in systemic sclerosis: ultrasound parameters and association with clinical manifestations. Arthritis Res Ther 23:122. 10.1186/s13075-021-02505-y 33882993 10.1186/s13075-021-02505-y PMC8059267

[35] Shan Y, Lee M, Chang EB (2022) The gut microbiome and inflammatory bowel diseases. Annu Rev Med 73:455–468. 10.1146/annurev-med-042320-021020 34555295 10.1146/annurev-med-042320-021020 PMC10012812

[36] Stec A, Maciejewska M, Zaremba M et al (2023) The clinical significance of serum biomarkers of the intestinal barrier in systemic sclerosis: a cross-sectional study. J Pers Med 13:678. 10.3390/jpm13040678 37109064 10.3390/jpm13040678 PMC10141873

[37] Kawagachi Y, Kuwana M (2023) Is there a role for the microbiome in systemic sclerosis? Expert Rev Clin Immunol 19:237–240. 10.1080/1744666X.2023.2161512 36576305 10.1080/1744666X.2023.2161512 PMC9957839

[38] Bramer WM (2018) A systemic approach to searching: an efficient and complete method to develop literature searches. J Med Libr Assoc 106(4):531–541. 10.5195/jmla.2018.283 30271302 10.5195/jmla.2018.283 PMC6148622

[39] Horvath AR, Lord SJ, StJohn A et al (2014) From biomarkers to medical tests: the changing landscape of test evaluation. Clin Chim Acta 427:49–57. 10.1016/j.cca.2013.09.018 24076255 10.1016/j.cca.2013.09.018

[40] Stochmal A, Czuwara J, Trojanowska M, Rudnicka L (2020) Antinuclear antibodies in systemic sclerosis: an update. Clin Rev Allergy Immunol 58:40–51. 10.1007/s12016-018-8718-8 30607749 10.1007/s12016-018-8718-8

[41] Papaioannou AI, Zakynthinos E, Kostikas K et al (2009) Serum VEGF levels are related to the presence of pulmonary arterial hypertension in systemic sclerosis. BMC Pulm Med 9:18. 10.1186/1471-2466-9-18 19426547 10.1186/1471-2466-9-18 PMC2685370

[42] Rokni M, Sadeghi Shaker M, Kavosi H et al (2022) The role of endothelin and RAS/ERK signaling in immunopathogenesis-related fibrosis in patients with systemic sclerosis: an updated review with therapeutic implications. Arthritis Res Ther 24:108. 10.1186/s13075-022-02787-w 35562771 10.1186/s13075-022-02787-w PMC9102675

[43] Bellando-Randone S, Matucci-Cerinic M (2014) From Raynaud’s phenomenon to very early diagnosis of systemic sclerosis—the VEDOSS approach. Curr Rheumatol Rev 9:245–248. 10.2174/157339710904140417124819 10.2174/157339710904140417124819 26932288

[44] Patnaik E, Lyons M, Tran K, Pattanaik D (2023) Endotheial dysfunction in systemic sclerosis. Int J Mol Sci 24:14385. 10.3391/ijms241814385 37762689 10.3390/ijms241814385 PMC10531630

[45] Ho KT, Reveille JD (2003) The clinical relevance of autoantibodies in scleroderma. Arthritis Res Ther 5:80–93. 10.1186/ar628 12718748 10.1186/ar628 PMC165038

[46] Volpe A, Ruzzenente O, Caramaschi P et al (2009) Clinical associations of anti-CENP-B and anti-Scl70 antibody levels measured by multiplexed fluorescent microsphere immunoassay in systemic sclerosis. Rheumatol Int 29:1073–1079. 10.1007/s00296-009-0868-9 19194705 10.1007/s00296-009-0868-9

[47] Okano Y, Steen VD, Medsger TA (1993) Autoantibody reactive with RNA polymerase III in systemic sclerosis. Ann Intern Med 119:1005–1013. 10.7326/0003-4819-119-10-199311150-00007 8214977 10.7326/0003-4819-119-10-199311150-00007

[48] Mahler M, Meroni P-L, Bossuyt X, Fritzler MJ (2014) Current concepts and future directions for the assessment of autoantibodies to cellular antigens referred to as anti-nuclear antibodies. J Immunol Res 2014:1–18. 10.1155/2014/315179 10.1155/2014/315179 PMC4020446 24868563

[49] Betteridge ZE, Woodhead F, Lu H et al (2016) Brief report: anti–eukaryotic initiation factor 2B autoantibodies are associated with interstitial lung disease in patients With systemic sclerosis. Arthritis Rheumatol 68:2778–2783. 10.1002/art.39755 27273608 10.1002/art.39755

[50] Pauling JD, Salazar G, Lu H et al (2018) Presence of anti-eukaryotic initiation factor-2B, anti-RuvBL1/2 and anti-synthetase antibodies in patients with anti-nuclear antibody negative systemic sclerosis. Rheumatol (United Kingdom) 57:712–717. 10.1093/rheumatology/kex458 10.1093/rheumatology/kex458 29294089

[51] Fertig N, Domsic RT, Rodriguez-Reyna T et al (2009) Anti-U11/U12 RNP antibodies in systemic sclerosis: a new serologic marker associated with pulmonary fibrosis. Arthritis Care Res 61:958–965. 10.1002/art.24586 10.1002/art.24586 PMC2739404 19565553

[52] Wirtz D, Schulte-Pelkum J, Budde P, et al (2015) Development of a qualitative ELISA for the detection of anti-BICD2 autoantibodies in systemic sclerosis. In: 12th Dresden Symposium on Autoantibodies, P53. Dresden

[53] Chan EKL (2022) Anti-Ro52 autoantibody is common in systemic autoimmune rheumatic diseases and correlating with worse outcome when associated with interstitial lung disease in systemic sclerosis and autoimmune myositis. Clin Rev Allergy Immunol 63:178–193. 10.1007/s12016-021-08911-z 35040083 10.1007/s12016-021-08911-z

[54] Mahler M, Swart A, Wu J et al (2016) Clinical and serological associations of autoantibodies to the Ku70/Ku80 heterodimer determined by a novel chemiluminescent immunoassay. Lupus 25:889–896. 10.1177/0961203316640918 27252266 10.1177/0961203316640918

[55] Mahler M, Fritzler MJ (2009) The changing landscape of the clinical value of the PM/Scl autoantibody system. Arthritis Res Ther 11:106. 10.1186/ar2646 19351430 10.1186/ar2646 PMC2688186

[56] Airo’CeribelliCavazzana PAI et al (2011) Malignancies in Italian patients with systemic sclerosis positive for anti-RNA polymerase III antibodies. J Rheumatol 38:1329–1334. 10.3899/jrheum.101144 21459934 10.3899/jrheum.101144

[57] Moinzadeh P, Fonseca C, Hellmich M et al (2014) Association of anti-RNA polymerase III autoantibodies and cancer in scleroderma. Arthritis Res Ther 16:R53. 10.1186/ar4486 24524733 10.1186/ar4486 PMC3978927

[58] Xu GJ, Shah AA, Li MZ et al (2016) Systematic autoantigen analysis identifies a distinct subtype of scleroderma with coincident cancer. Proc Natl Acad Sci U S A 113:E7526–E7534. 10.1073/pnas.1615990113 27821747 10.1073/pnas.1615990113 PMC5127349

[59] Nakamura K, Asano Y, Taniguchi T et al (2016) Serum levels of interleukin-18-binding protein isoform a: clinical association with inflammation and pulmonary hypertension in systemic sclerosis. J Dermatol 43:912–918. 10.1111/1346-8138.13252 26777734 10.1111/1346-8138.13252

[60] Hasegawa M, Fujimoto M, Kikuchi K, Takehara K (1997) Elevated serum levels of interleukin 4 (IL-4), IL-10, and IL-13 in patients with systemic sclerosis. J Rheumatol 24:328–332 9034992

[61] Bălănescu P, Lădaru A, Bălănescu E et al (2015) IL-17, IL-6 and IFN-γ in systemic sclerosis patients. Rom J Intern Med 53:44–49. 10.1515/rjim-2015-0006 26076560 10.1515/rjim-2015-0006

[62] Robak E, Kulczycka-Siennicka L, Gerlicz Z et al (2013) Correlations between concentrations of interleukin (IL)-17A, IL-17B and IL-17F, and endothelial cells and proangiogenic cytokines in systemic lupus erythematosus patients. Eur Cytokine Netw 24:60–68. 10.1684/ecn.2013.0330 23661335 10.1684/ecn.2013.0330

[63] Xu B, Xu G, Yu Y, Lin J (2021) The role of TGF-β or BMPR2 signaling pathway-related miRNA in pulmonary arterial hypertension and systemic sclerosis. Arthritis Res Ther 23:288. 10.1186/s13075-021-02678-6 34819148 10.1186/s13075-021-02678-6 PMC8613994

[64] Bukiri H, Volkmann ER (2022) Current advances in the treatment of systemic sclerosis. Curr Opin Pharmacol 64:102211. 10.1016/j.coph.2022.102211 35447517 10.1016/j.coph.2022.102211 PMC9466985

[65] Khanna D, Lin CJF, Furst DE et al (2022) Long-term safety and efficacy of tocilizumab in early systemic sclerosis–interstitial lung disease: open-label extension of a phase 3 randomized controlled trial. Am J Respir Crit Care Med 205:674–684. 10.1164/rccm.202103-0714OC 34851799 10.1164/rccm.202103-0714OC

[66] O’Reilly S, Cant R, Ciechkomaka M, van Laar JM (2013) Interleukin-6: a new therapeutic target in systemic sclerosis? Clin Transl Immunol 2. 10.1038/cti.2013.2 10.1038/cti.2013.2 PMC4232056 25505952

[67] Jalce G, Guignabert C (2020) Multiple roles of macrophage migration inhibitory factor in pulmonary hypertension. Am J Physiol Cell Mol Physiol 318:L1–L9. 10.1152/ajplung.00234.2019 10.1152/ajplung.00234.2019 31577159

[68] Frantz C, Cauvet A, Durand A et al (2022) Driving role of interleukin-2–related regulatory CD4+ T cell deficiency in the development of lung fibrosis and vascular remodeling in a mouse model of systemic sclerosis. Arthritis Rheumatol 74:1387–1398. 10.1002/art.42111 35255201 10.1002/art.42111

[69] Kosałka-Węgiel J, Lichołai S, Dziedzina S et al (2022) Genetic association between TNFA polymorphisms (rs1799964 and rs361525) and susceptibility to cancer in systemic sclerosis. Life 12:698. 10.3390/life12050698 35629365 10.3390/life12050698 PMC9145848

[70] Lykhopiy V, Malviya V, Humblet-Baron S, Schlenner SM (2023) IL-immunotherapy for targeting regulatory T cells in autoimmunity. Genes Immun 24:248–262. 10.1038/s41435-023-00221-y 37741949 10.1038/s41435-023-00221-y PMC10575774

[71] King J, Abraham D, Stratton R (2018) Chemokines in systemic sclerosis. Immunol Lett 195:68–75. 10.1016/j.imlet.2017.12.001 29247681 10.1016/j.imlet.2017.12.001

[72] Khanna D, Tashkin DP, Denton CP et al (2020) Etiology, risk factors, and biomarkers in systemic sclerosis with interstitial lung disease. Am J Respir Crit Care Med 201:650–660. 10.1164/rccm.201903-0563CI 31841044 10.1164/rccm.201903-0563CI PMC7068837

[73] Furukawa T, Matsui K, Kitano M et al (2019) Relationship between YKL-40 and pulmonary arterial hypertension in systemic sclerosis. Mod Rheumatol 29:476–483. 10.1080/14397595.2018.1480256 29788800 10.1080/14397595.2018.1480256

[74] Machahua C, Guler SA, Horn MP et al (2021) Serum calprotectin as new biomarker for disease severity in idiopathic pulmonary fibrosis: a cross-sectional study in two independent cohorts. BMJ Open Respir Res 8:e000827. 10.1136/bmjresp-2020-000827 33451989 10.1136/bmjresp-2020-000827 PMC7813379

[75] Boleto G, Allanore Y, Avouac J (2018) Targeting costimulatory pathways in systemic sclerosis. Front Immunol 9:2998. 10.3389/fimmu.2018.02998 30619351 10.3389/fimmu.2018.02998 PMC6305435

[76] Romano E, Rosa I, Fioretto BS et al (2019) A new avenue in the pathogenesis of systemic sclerosis: the molecular interface between the endothelial and the nervous systems. Clin Exp Rheumatol 37(Suppl 1):133–140 31025932

[77] Romano E, Rosa I, Fioretto BS et al (2022) Circulating neurovascular guidance molecules and their relationship with peripheral microvascular impairment in systemic sclerosis. Life 12:1056. 10.3390/life12071056 35888144 10.3390/life12071056 PMC9316343

[78] Mazzotta C, Romano E, Bruni C et al (2015) Plexin-D1/Semaphorin 3E pathway may contribute to dysregulation of vascular tone control and defective angiogenesis in systemic sclerosis. Arthritis Res Ther 17:221. 10.1186/s13075-015-0749-4 26292963 10.1186/s13075-015-0749-4 PMC4546224

[79] Michalska-Jakubus M, Potembska E, Kowal M et al (2019) Clinical associations of serum leptin and leptin/adiponectin ratio in systemic sclerosis. Postep Dermatologii Alergol 36:325–338. 10.5114/ada.2018.75809 10.5114/ada.2018.75809 PMC6640022 31333350

[80] Pehlivan Y, Onat AM, Ceylan N et al (2012) Serum leptin, resistin and TNF-α levels in patients with systemic sclerosis: the role of adipokines in scleroderma. Int J Rheum Dis 15:374–379. 10.1111/j.1756-185X.2012.01755.x 22898217 10.1111/j.1756-185X.2012.01755.x

[81] Sundblad V, Gomez RA, Stupirski JC et al (2021) Circulating galectin-1 and galectin-3 in sera from patients with systemic sclerosis: associations with clinical features and treatment. Front Pharmacol 12:650605. 10.3389/fphar.2021.650605 33959016 10.3389/fphar.2021.650605 PMC8093796

[82] Miura S, Asano Y, Saigusa R et al (2015) Serum vaspin levels: a possible correlation with digital ulcers in patients with systemic sclerosis. J Dermatol 42:528–531. 10.1111/1346-8138.12810 25708680 10.1111/1346-8138.12810

[83] Sawicka K, Michalska-Jakubus M, Potembska E et al (2019) Visfatin and chemerin levels correspond with inflammation and might reflect the bridge between metabolism, inflammation and fibrosis in patients with systemic sclerosis. Postep Dermatologii i Alergol 36:551–565. 10.5114/ada.2018.79104 10.5114/ada.2018.79104 PMC6906965 31839772

[84] Sanges S, Rice L, Tu L et al (2023) Biomarkers of haemodynamic severity of systemic sclerosis-associated pulmonary arterial hypertension by serum proteome analysis. Ann Rheum Dis 82:365–373. 10.1136/ard-2022-223237 36600187 10.1136/ard-2022-223237 PMC9918672

[85] Hasegawa M, Asano Y, Endo H et al (2014) Serum adhesion molecule levels as prognostic markers in patients with early systemic sclerosis: a multicentre, prospective, observational study. PLoS One 6(9):e88150. 10.1371/journal.pone.0088150. (**eCollection 2014**) 10.1371/journal.pone.0088150 PMC3916412 24516598

[86] Kuszmiersz P, Pacholczak-Madej R, Siwiec A et al (2021) Thrombin generation potential is enhanced in systemic sclerosis: impact of selected endothelial biomarkers. Clin Exp Rheumatol 131:13–19. 10.55563/clinexprheumatol/d03dnc 10.55563/clinexprheumatol/d03dnc 33769265

[87] Ren H, Liu L, Xiao Y et al (2023) Further insight into systemic sclerosis from the vasculopathy perspective. Biomed Pharmacother. 10.1016/j.biopha.2023.115282 37567070 10.1016/j.biopha.2023.115282

[88] Gigante A, NavariniMargiotta LD et al (2017) Angiogenic and angiostatic factors in renal scleroderma-associated vasculopathy. Microvasc Res 114:41–45. 10.1016/j.mvr.2017.06.003 28602918 10.1016/j.mvr.2017.06.003

[89] Luo JY, Liu X, Jiang M et al (2017) Oxidative stress markers in blood in systemic sclerosis: a meta-analysis. Mod Rheumatol 27:306–314. 10.1080/14397595.2016.1206510 27425641 10.1080/14397595.2016.1206510

[90] Doridot L, Jeljeli M, Chệne C, Batteux F (2019) Implication of oxidative stress in the pathogenesis of systemic sclerosis via inflammation, autoimmunity and fibrosis. Redox Biol 25:101122. 10.1016/j.redox.2019.101122 30737171 10.1016/j.redox.2019.101122 PMC6859527

[91] Cumpstey A, Feelisch M (2017) Free radicals in inflammation. Inflammation—from molecular and cellular mechanisms to the clinic. Wiley-VCH Verlag GmbH & Co. KGaA, Weinheim, Germany, pp 695–726

[92] Chettimada S, Ata H, Rawat DK et al (2014) Contractile protein expression is upregulated by reactive oxygen species in aorta of Goto-Kakizaki rat. Am J Physiol Circ Physiol 306:H214–H224. 10.1152/ajpheart.00310.2013 10.1152/ajpheart.00310.2013 PMC3920128 24213617

[93] Dimmeler S, Zeiher AM (2000) Reactive oxygen species and vascular cell apoptosis in response to angiotensin II and pro-atherosclerotic factors. Regul Pept 90:19–25. 10.1016/S0167-0115(00)00105-1 10828488 10.1016/s0167-0115(00)00105-1

[94] Cortese-Krott MM, Koning A, Kuhnle GGC et al (2017) The reactive species interactome: evolutionary emergence, biological significance, and opportunities for redox metabolomics and personalized medicine. Antioxid Redox Signal 27:684–712. 10.1089/ars.2017.7083 28398072 10.1089/ars.2017.7083 PMC5576088

[95] Maxwell PH, Ratcliffe PJ (2002) Oxygen sensors and angiogenesis. Semin Cell Dev Biol 13:29–37. 10.1006/scdb.2001.0287 11969369 10.1006/scdb.2001.0287

[96] Ke Q, Costa M (2006) Hypoxia-inducible factor-1 (HIF-1). Mol Pharmacol 70:1469–1480. 10.1124/mol.106.027029 16887934 10.1124/mol.106.027029

[97] Distler O, Distler JHW, Scheid A et al (2004) Uncontrolled expression of vascular endothelial growth factor and its receptors leads to insufficient skin angiogenesis in patients with systemic sclerosis. Circ Res 95:109–116. 10.1161/01.RES.0000134644.89917.96 15178641 10.1161/01.RES.0000134644.89917.96

[98] Grunewald M, Avraham I, Dor Y et al (2006) VEGF-induced adult neovascularization: recruitment, retention, and role of accessory cells. Cell 124:175–189. 10.1016/j.cell.2005.10.036 16413490 10.1016/j.cell.2005.10.036

[99] Distler JHW, Jüngel A, Pileckyte M et al (2007) Hypoxia-induced increase in the production of extracellular matrix proteins in systemic sclerosis. Arthritis Rheum 56:4203–4215. 10.1002/art.23074 18050252 10.1002/art.23074

[100] Falanga V, Tiegs SL, Alstadt SP et al (1987) Transforming growth factor-beta: Selective increase in glycosaminoglycan synthesis by cultures of fibroblasts from patients with progressive systemic sclerosis. J Invest Dermatol 89:100–104. 10.1111/1523-1747.ep12580445 3496398 10.1111/1523-1747.ep12580445

[101] Bertolotti M, Sitia R, Rubartelli A (2012) On the redox control of B lymphocyte differentiation and function. Antioxid Redox Signal 16:1139–1149. 10.1089/ars.2011.4252 22229488 10.1089/ars.2011.4252

[102] O’Reilly S, Hugle T, van Laar JM (2012) T cells in systemic sclerosis: a reappraisal. Rheumatology 51:1540–1549. 10.1093/rheumatology/kes090 22577083 10.1093/rheumatology/kes090

[103] Mo C, Zeng Z, Deng Q et al (2018) Imbalance between T helper 17 and regulatory T cell subsets plays a significant role in the pathogenesis of systemic sclerosis. Biomed Pharmacother 108:177–183. 10.1016/j.biopha.2018.09.037 30219674 10.1016/j.biopha.2018.09.037

[104] Abais JM, Xia M, Zhang Y et al (2015) Redox regulation of NLRP3 inflammasomes: ROS as trigger or effector? Antioxid Redox Signal 22:1111–1129. 10.1089/ars.2014.5994 25330206 10.1089/ars.2014.5994 PMC4403231

[105] Taroni JN, Greene CS, Martyanov V et al (2017) A novel multi-network approach reveals tissue-specific cellular modulators of fibrosis in systemic sclerosis. Genome Med 9:27. 10.1186/s13073-017-0417-1 28330499 10.1186/s13073-017-0417-1 PMC5363043

[106] Dantas AT, Gonçalves SMC, De AAR et al (2016) Reassessing the role of the active TGF- β 1 as a biomarker in systemic sclerosis: association of serum levels with clinical manifestations. Dis Markers 2016:1–6. 10.1155/2016/6064830 10.1155/2016/6064830 PMC5124685 27965520

[107] Meng C, Chen X, Li J et al (2008) Expression of MMP-9 and TIMP-1 in lesions of systemic sclerosis and its implications. J Huazhong Univ Sci Technol - Med Sci 28:480–482. 10.1007/s11596-008-0424-y 10.1007/s11596-008-0424-y 18704317

[108] Manetti M, Guiducci S, Romano E et al (2012) Increased serum levels and tissue expression of matrix metalloproteinase-12 in patients with systemic sclerosis: correlation with severity of skin and pulmonary fibrosis and vascular damage. Ann Rheum Dis 71:1064–1072. 10.1136/annrheumdis-2011-200837 22258486 10.1136/annrheumdis-2011-200837

[109] Asano Y, Ihn H, Kubo M et al (2006) Clinical significance of serum levels of matrix metalloproteinase-13 in patients with systemic sclerosis. Rheumatology 45:303–307. 10.1093/rheumatology/kei143 16278285 10.1093/rheumatology/kei143

[110] Casciola-Rosen L, Wigley F, Rosen A (1997) Scleroderma autoantigens are uniquely fragmented by metal-catalyzed oxidation reactions: implications for pathogenesis. J Exp Med 185:71–79. 10.1084/jem.185.1.71 8996243 10.1084/jem.185.1.71 PMC2196102

[111] Iwata Y, Ogawa F, Komura K et al (2007) Autoantibody against peroxiredoxin I, an antioxidant enzyme, in patients with systemic sclerosis: possible association with oxidative stress. Rheumatology 46:790–795. 10.1093/rheumatology/kem010 17309887 10.1093/rheumatology/kem010

[112] Ogawa F, Shimizu K, Hara T et al (2010) Autoantibody against one of the antioxidant repair enzymes, methionine sulfoxide reductase A, in systemic sclerosis: association with pulmonary fibrosis and vascular damage. Arch Dermatol Res 302:27–35. 10.1007/s00403-009-0996-9 19844733 10.1007/s00403-009-0996-9

[113] Dziedzic R, Wójcik K, Olchawa M et al (2023) Increased oxidative stress response in circulating blood of systemic sclerosis patients—relation to disease characteristics and inflammatory blood biomarkers. Semin Arthritis Rheum 62:152228. 10.1016/j.semarthrit.2023.152228 37429138 10.1016/j.semarthrit.2023.152228

[114] McMahan ZH, Wigley FM, Casciola-Rosen L (2017) Risk of digital vascular events in scleroderma patients who have both anticentromere and anti–interferon-inducible protein 16 antibodies. Arthritis Care Res 69:922–926. 10.1002/acr.22978 10.1002/acr.22978 PMC5219877 27389713

[115] Gigante A, Margiotta D, Navarini L et al (2018) Serum level of endostatin and digital ulcers in systemic sclerosis patients. Int Wound J 15:424–428. 10.1111/iwj.12882 29600562 10.1111/iwj.12882 PMC7950077

[116] Schiopu E, Au KM, McMahon MA et al (2014) Prevalence of subclinical atherosclerosis is increased in systemic sclerosis and is associated with serum proteins: a cross-sectional, controlled study of carotid ultrasound. Rheumatology 53:704–713. 10.1093/rheumatology/ket411 24357811 10.1093/rheumatology/ket411 PMC4042927

[117] Habe K, Wada H, Higashiyama A et al (2018) The plasma levels of ADAMTS-13, von Willebrand factor, VWFpp, and fibrin-related markers in patients with systemic sclerosis having thrombosis. Clin Appl Thromb 24:920–927. 10.1177/1076029617736382 10.1177/1076029617736382 PMC6714713 29130325

[118] Kovacs G, Olschewski H (2019) Potential role of exercise echocardiography and right heart catheterization in the detection of early pulmonary vascular disease in patients with systemic sclerosis. J Scleroderma Relat Disord 4:219–224. 10.1177/2397198319849805 35382501 10.1177/2397198319849805 PMC8922565

[119] Elshamy HA, Ibrahim SE, Farouk HM et al (2011) N-Terminal pro-brain natriuretic peptide in systemic sclerosis: new insights. Eur J Dermatology 21:686–690. 10.1684/ejd.2011.1423 10.1684/ejd.2011.1423 21700537

[120] Jha M, Wang M, Steele R et al (2022) NT-proBNP, hs-cTnT, and CRP predict the risk of cardiopulmonary outcomes in systemic sclerosis: findings from the Canadian Scleroderma Research Group. J Scleroderma Relat Disord 7:62–70. 10.1177/23971983211040608 35386945 10.1177/23971983211040608 PMC8922674

[121] Kill A, Tabeling C, Undeutsch R et al (2014) Autoantibodies to angiotensin and endothelin receptors in systemic sclerosis induce cellular and systemic events associated with disease pathogenesis. Arthritis Res Ther 16:R29. 10.1186/ar4457 24472528 10.1186/ar4457 PMC3978438

[122] Lammi MR, Saketkoo LA, Okpechi SC et al (2019) Microparticles in systemic sclerosis: potential pro-inflammatory mediators and pulmonary hypertension biomarkers. Respirology 24:675–683. 10.1111/resp.13500 30747487 10.1111/resp.13500 PMC6579687

[123] Panse KD, Felkin LE, López-Olañeta MM et al (2012) Follistatin-like 3 mediates paracrine fibroblast activation by cardiomyocytes. J Cardiovasc Transl Res 5:814–826. 10.1007/s12265-012-9400-9 22915069 10.1007/s12265-012-9400-9

[124] Izumiya Y, Jinnn M, Kimura Y et al (2015) Expression of Let-7 family microRNAs in skin correlates negatively with severity of pulmonary hypertension in patients with systemic scleroderma. IJC Hear Vasc 8:98–102. 10.1016/j.ijcha.2015.06.006 10.1016/j.ijcha.2015.06.006 PMC5497286 28785688

[125] Sun Q, Hackler J, Hilger J et al (2020) Selenium and copper as biomarkers for pulmonary arterial hypertension in systemic sclerosis. Nutrients 12:1–13. 10.3390/nu12061894 10.3390/nu12061894 PMC7353414 32630589

[126] Perelas A, Silver RM, Arrossi AV, Highland KB (2020) Systemic sclerosis-associated interstitial lung disease. Lancet Respir Med 8:304–320. 10.1016/S2213-2600(19)30480-1 32113575 10.1016/S2213-2600(19)30480-1

[127] Bütikofer L, Varisco PA, Distler O et al (2020) ACE inhibitors in SSc patients display a risk factor for scleroderma renal crisis—a EUSTAR analysis. Arthritis Res Ther 22:59. 10.1186/s13075-020-2141-2 32209135 10.1186/s13075-020-2141-2 PMC7093969

[128] Torrisi SE, Palmucci S, Stefano A et al (2018) Assessment of survival in patients with idiopathic pulmonary fibrosis using quantitative HRCT indexes. Multidiscip Respir Med 13:43. 10.1186/s40248-018-0155-2 30519466 10.1186/s40248-018-0155-2 PMC6271409

[129] Moradzadeh M, Aghaei M, Mehrbakhsh Z et al (2021) Efficacy and safety of rituximab therapy in patients with systemic sclerosis disease (SSc): systematic review and meta-analysis. Clin Rheumatol 40:3897–3918. 10.1007/s10067-021-05698-4 33796953 10.1007/s10067-021-05698-4

[130] Mouawaid JE, Feghali-Bostwick C (2023) The molecular mechanism of systemic sclerosis-associated lung fibrosis 24:2963. 10.3390/ijms24032963 10.3390/ijms24032963 PMC9917655 36769282

[131] Decker P, Moulinet T, Lopez B et al (2021) Clinical significance of anti-Ro52 (TRIM21) antibodies in adult patients with connective tissue diseases. Eur J Intern Med 91:45–52. 10.1016/j.ejim.2021.04.020 33972152 10.1016/j.ejim.2021.04.020

[132] Lee JS, Lee EY, Ha Y-J et al (2019) Serum KL-6 levels reflect the severity of interstitial lung disease associated with connective tissue disease. Arthritis Res Ther 21:58. 10.1186/s13075-019-1835-9 30764869 10.1186/s13075-019-1835-9 PMC6376648

[133] Leong E, Bezuhly M, Marshall JS (2021) Distinct metalloproteinase expression and functions in systemic sclerosis and fibrosis: what we know and the potential for intervention. Front Physiol 12(12):72745. 10.3389/fphys.2021.727451 10.3389/fphys.2021.727451 PMC8432940 34512395

[134] Weigold F, Günther J, Pfeiffenberger M et al (2018) Antibodies against chemokine receptors CXCR3 and CXCR4 predict progressive deterioration of lung function in patients with systemic sclerosis. Arthritis Res Ther 20:52. 10.1186/s13075-018-1545-8 29566745 10.1186/s13075-018-1545-8 PMC5863842

[135] Dichev V, Mehterov NH, Kazakova MH et al (2021) Serum protein levels of YKL-40 and plasma miR-214 expression in patients with systemic sclerosis. Mod Rheumatol 31:1010–1018. 10.1080/14397595.2020.1859726 33274678 10.1080/14397595.2020.1859726

[136] Suga M, Iyonaga K, Ichiyasu H et al (1999) Clinical significance of MCP-1 levels in BALF and serum in patients with interstitial lung diseases. Eur Respir J 14:376–382. 10.1034/j.1399-3003.1999.14b23.x 10515417 10.1034/j.1399-3003.1999.14b23.x

[137] Grosicka A, Manasar A, Kucharz EJ, Kotyla PJ (2018) Serum concentration of surfactant protein D in patients with systemic sclerosis: the potential marker of the interstitial lung disease severity. Best Pract Res Clin Rheumatol 32:541–549. 10.1016/j.berh.2019.01.005 31174823 10.1016/j.berh.2019.01.005

[138] Wang K, Ju Q, Cao J et al (2017) Impact of serum SP-A and SP-D levels on comparison and prognosis of idiopathic pulmonary fibrosis. Medicine (Baltimore) 96:e7083. 10.1097/MD.0000000000007083 28591049 10.1097/MD.0000000000007083 PMC5466227

[139] Elhai M, Avouac J, Hoffmann-Vold AM et al (2016) OX40L blockade protects against inflammationdriven fibrosis. Proc Natl Acad Sci U S A 113:E3901–E3910. 10.1073/pnas.1523512113 27298374 10.1073/pnas.1523512113 PMC4941508

[140] Żółkiewicz J, Stochmal A, Rudnicka L (2019) The role of adipokines in systemic sclerosis: a missing link? Arch Dermatol Res 311:251–263. 10.1007/s00403-019-01893-1 30806766 10.1007/s00403-019-01893-1 PMC6469644

[141] Kawabata K, Makino T, Makino K et al (2020) IL-16 expression is increased in the skin and sera of patients with systemic sclerosis. Rheumatol (United Kingdom) 59:519–523. 10.1093/rheumatology/kez318 10.1093/rheumatology/kez318 31377804

[142] Leodori G, Pellicano C, Basile V et al (2022) Serum adiponectin, a novel biomarker correlates with skin thickness in systemic sclerosis. J Pers Med 12:1737. 10.3390/jpm12101737 36294874 10.3390/jpm12101737 PMC9604668

[143] Farina G, Lafyatis D, Lemaire R, Farina LG, R, (2010) A four-gene biomarker predicts skin disease in patients with diffuse cutaneous systemic sclerosis. Arthritis Rheum 62:580–588. 10.1002/art.27220 20112379 10.1002/art.27220 PMC3018285

[144] Stern EP, Guerra SG, Chinque H et al (2020) Analysis of anti-RNA polymerase III antibody-positive systemic sclerosis and altered GPATCH2L and CTNND2 expression in scleroderma renal crisis. J Rheumatol 47:1668–1677. 10.3899/jrheum.190945 32173657 10.3899/jrheum.190945

[145] Okrój M, Johansson M, Saxne T et al (2016) Analysis of complement biomarkers in systemic sclerosis indicates a distinct pattern in scleroderma renal crisis. Arthritis Res Ther 18:267. 10.1186/s13075-016-1168-x 27863511 10.1186/s13075-016-1168-x PMC5116178

[146] Nguyen AD, Andréasson K, McMahan ZH et al (2023) Gastrointestinal tract involvement in systemic sclerosis: the roles of diet and the microbiome. Semin Arthritis Rheum 60:152185. 10.1016/j.semarthrit.2023.152185 36870237 10.1016/j.semarthrit.2023.152185 PMC10148899

[147] Andréasson K, Scheja A, Saxne T et al (2011) Faecal calprotectin: a biomarker of gastrointestinal disease in systemic sclerosis. J Intern Med 270:50–57. 10.1111/j.1365-2796.2010.02340.x 21205026 10.1111/j.1365-2796.2010.02340.x

[148] Goldblatt F, Gordon TP, Waterman SA (2002) Antibody-mediated gastrointestinal dysmotility in scleroderma. Gastroenterology 123:1144–1150. 10.1053/gast.2002.36057 12360477 10.1053/gast.2002.36057

[149] Hamberg V, Wallman JK, Mogard E et al (2023) Elevated fecal levels of the inflammatory biomarker calprotectin in early systemic sclerosis. Rheumatol Int 43:961–967. 10.1007/s00296-022-05264-4 36566433 10.1007/s00296-022-05264-4 PMC10073054

[150] Weeding E, Casciola-Rosen L, Shah AA (2020) Cancer and scleroderma. Rheum Dis Clin North Am 46:551–564. 10.1016/j.rdc.2020.03.002 32631603 10.1016/j.rdc.2020.03.002 PMC7340850

[151] Guillen-Del-Castillo A, Simeón-Aznar CP (2023) Identifying the risk of cancer-associated systemic sclerosis. Joint Bone Spine 90:105618. 10.1016/j.jbspin.2023.105618 37482176 10.1016/j.jbspin.2023.105618

[152] Onishi A, Sugiyama D, Kumagai S, Morinobu A (2013) Cancer incidence in systemic sclerosis: meta-analysis of population-based cohort studies. Arthritis Rheum 65:1913–1921. 10.1002/art.37969 23576072 10.1002/art.37969

[153] Sargin G, Senturk T, Cildag S (2018) Systemic sclerosis and malignancy. Int J Rheum Dis 21:1093–1097. 10.1111/1756-185X.13311 29673080 10.1111/1756-185X.13311

[154] Hoa S, Lazizi S, Baron M et al (2022) Association between autoantibodies in systemic sclerosis and cancer in a national registry. Rheumatology (Oxford) 61:2905–2914. 10.1093/rheumatology/keab735 34599801 10.1093/rheumatology/keab735

[155] Di Battista M, Lepri G, Codullo V et al (2023) Systemic sclerosis: one year in review. Clin Exp Rheumatol 41:1567–1574. 10.55563/clinexperheumatol/ki76s5 37199215 10.55563/clinexprheumatol/ki76s5

[156] Hummers LK (2023) Clinical trials in systemic sclerosis: crossroads and opportunities. Arthritis Rehumatol 75:1328–1330. 10.1002/art.42524 10.1002/art.42514 37011044

[157] Campochiaro C, Allanore Y (2021) An update on targeted therapies in systemic sclerosis based on a systematic review from the last 3 years. Arthritis Res Ther 23:155. 10.1186/s13075-021-02536-5 34074331 10.1186/s13075-021-02536-5 PMC8168022

[158] Khanna D, Kramer F, Höfler J et al (2024) Biomarker analysis from the phase 2b randomized placebo-controlled trial of riociguat in early diffuse cutaneous systemic sclerosis. Rheumatology (Oxford) 9:keae150. 10.1093/rheumatology/keae150 10.1093/rheumatology/keae150 PMC11534119 38460548

