# Correction: Biomarkers reflecting the pathogenesis, clinical manifestations, and guide therapeutic approach in systemic sclerosis: a narrative review

**DOI:** 10.1007/s10067-024-07147-4

**Published:** 2024-09-25

**Authors:** Anna Bazsó, Péter Szodoray, Yehuda Shoenfeld, Emese Kiss

**Affiliations:** 1 Department of Clinical Immunology, Adult and Paediatric Rheumatology, National Institute of Locomotor System Disorders and Disabilities, Budapest, Hungary; 2 Department of Immunology, Oslo University Hospital, Rikshospitalet and University of Oslo, Oslo, Norway; 3 Reichmann University, Herzelia, Israel; 4 Zabludowicz Center for Autoimmune Diseases, Sheba Medical Center, 5265601 Tel-Hashomer, Israel; 5 Division of Locomotor System and Rheumatology Prevention, Department of Internal Medicine and Haematology, Semmelweis University, Budapest, Hungary

**Correction: Clinical Rheumatology**

10.1007/s10067-024-07123-y

In this article, the academic title "Med Dsci" for authors Péter Szodoray and Emese Kiss were captured.

The original article has been corrected.

